# Comprehensive landscape of m6A regulator-related gene patterns and tumor microenvironment infiltration characterization in gastric cancer

**DOI:** 10.1038/s41598-024-66744-0

**Published:** 2024-07-16

**Authors:** Bin Peng, Yinglin Lin, Gao Yi, Mingzhen Lin, Yao Xiao, Yezhenghong Qiu, Wenxia Yao, Xinke Zhou, Zhaoyu Liu

**Affiliations:** Key Laboratory of Biological Targeting Diagnosis, Therapy and Rehabilitation of Guangdong Higher Education Institutes, State Key Laboratory of Respiratory Disease, The Fifth Affiliated Hospital, Guangzhou Medical University, The Fifth Clinical College of Guangzhou Medical University, Guangzhou, China

**Keywords:** N6-methyladenosine (m^6^a) modification, m^6^A regulator-related pattern, m^6^A-related score, Gastric cancer, Tumor microenvironment, Immunotherapy, Cancer, Immunology, Microbiology, Biomarkers

## Abstract

The epigenetic regulation of N6-methyladenosine (m^6^A) has attracted considerable interest in tumor research, but the potential roles of m^6^A regulator-related genes, remain largely unknown within the context of gastric cancer (GC) and tumor microenvironment (TME). Here, a comprehensive strategy of data mining and computational biology utilizing multiple datasets based on 28 m^6^A regulators (including novel anti-readers) was employed to identify m^6^A regulator-related genes and patterns and elucidate their underlying mechanisms in GC. Subsequently, a scoring system was constructed to evaluate individual prognosis and immunotherapy response. Three distinct m^6^A regulator-related patterns were identified through the unsupervised clustering of 56 m^6^A regulator-related genes (all significantly associated with GC prognosis). TME characterization revealed that these patterns highly corresponded to immune-inflamed, immune-excluded, and immune-desert phenotypes, and their TME characteristics were highly consistent with different clinical outcomes and biological processes. Additionally, an m^6^A-related scoring system was developed to quantify the m^6^A modification pattern of individual samples. Low scores indicated high survival rates and high levels of immune activation, whereas high scores indicated stromal activation and tumor malignancy. Furthermore, the m^6^A-related scores were correlated with tumor mutation loads and various clinical traits, including molecular or histological subtypes and clinical stage or grade, and the score had predictive values across all digestive system tumors and even in all tumor types. Notably, a low score was linked to improved responses to anti-PD-1/L1 and anti-CTLA4 immunotherapy in three independent cohorts. This study has expanded the important role of m^6^A regulator-related genes in shaping TME diversity and clinical/biological traits of GC. The developed scoring system could help develop more effective immunotherapy strategies and personalized treatment guidance.

## Introduction

Gastric cancer (GC) is a highly prevalent tumor, ranking fifth with an incidence rate of 5.6% among all cancers^1^. Most patients with GC are diagnosed at an advanced stage, due to its asymptomatic nature^2^. Current treatment options for GC mainly include surgery, chemotherapy, radiotherapy, and immunotherapy^3,4^. Immunotherapy, especially immune checkpoint inhibitors (ICIs), is emerging as the first-line treatment of advanced or metastatic GC^5^. The immunological landscape within the tumor microenvironment (TME) plays a critical role in selecting appropriate immunotherapy strategies and predicting treatment outcomes. Tumors can be immunologically categorized as immune-inflamed, immune-desert, or immune-excluded phenotypes according to the extent of immune cell infiltration and activation status^6^. An immune-inflamed phenotype, characterized by robust immune cell infiltration and activated immunity, is associated with a favorable response to immunotherapy. On the other hand, poor infiltration is indicative of an immune-desert phenotype while a high density of immune cells at the tumor margin without infiltration corresponds to an immune-excluded phenotype^6^. Both immune-desert and immune-excluded phenotypes are associated with low response to immunotherapy and poor clinical outcomes^7,8^. These three categories are designed as clinically relevant classifiers, with “hot” representing the immune-inflamed phenotype and “cold” corresponding to both immune-desert phenotype and immune-excluded phenotype^9,10^. In summary, the TME is closely related to the selection of immunotherapy strategies and immunotherapeutic efficacy, and accurately assessing the exact immune phenotype within the microenvironment can offer clinical benefits.

N6-methyladenosine (m^6^A) modification is the most prevalent internal RNA modification in eukaryotes and plays a pivotal role in regulating post-transcriptional gene modification^11–13^. m^6^A modification is regulated by a group of m^6^A regulators, including “writers,” “erasers,” “readers,” and “anti-readers.” The writers, which are methyltransferases, include *METTL3*, *RBM15*, *METTL14*, *ZC3H13*, *WTAP*, and *CBLL1*. Conversely, the erasers, which function as demethyltransferases, comprise *ALKBH5* and *FTO*. Together, they maintain the dynamic balance of m^6^A modification^14^. A diverse array of RNA-binding proteins named “readers” include *HNRNPA2B*, *YTHDC1/2*, *YTHDF1/2/3*, *LRPPRC*, and *FMR1* and play a crucial role in the recognition of m^6^A motifs^15^. Additionally, novel regulators, such as *EWSR1*, *G3BP1*, and *LIN28A*, act as anti-readers during m^6^A modification on RNA, consequently diminishing the efficiency of protein-RNA binding^16,17^. m^6^A regulators are involved in various crucial biological processes, such as tissue development, spermatogenesis, cell proliferation, and stem cell differentiation^18–20^. The aberrant expression of m^6^A regulators and/or genetic changes are closely associated with the occurrence, metastasis, and chemo- and radio-resistance of multiple malignant tumors, including GC, lung cancer, and hepatocellular carcinoma^21,22^. Moreover, m^6^A regulators play a crucial role in TME regulation. For instance, *FTO*, the first known m^6^A demethyltransferase, is closely related to TME remodeling and tumor escape^23,24^, and *FTO* inhibitors exhibit strong antitumor effects in various types of cancer^25^. A combination of treatment with *FTO* inhibitors and anti-PD-1 therapy exhibits good clinical efficacy in patients with melanoma^26^.

The pivotal roles of m^6^A regulators in TME regulation have attracted extensive attention^27,28^. Additionally, m^6^A regulator-related noncoding RNAs (ncRNAs), especially long noncoding RNAs (lncRNAs), have attracted considerable research. Emerging lines of studies have uncovered the clinical relevance of m^6^A regulator-related lncRNAs in various tumors, such as GC, lung adenocarcinoma, colon adenocarcinoma, breast cancer, and bladder cancer, and these lncRNA signatures exhibit excellent clinical applicability in tumor diagnosis and prognosis^29–32^. Furthermore, investigations demonstrate a close correlation between m^6^A regulator-related lncRNA signatures and TME, potentially predicting the efficacy of tumor immunotherapy^31,33,34^. However, the role of m^6^A regulator-related coding genes in tumors is rarely reported, despite their fundamental contributions to nearly all biological processes. In this investigation, we aim to comprehensively evaluate the m^6^A regulator-related patterns, derived from both coding genes and ncRNA genes, as well as their correlation with the immune cell infiltrating landscape of the TME. We have identified three distinct m^6^A regulator-related patterns, and found that the TME traits of these patterns were highly consistent with the immune-inflamed, immune-excluded, and immune-desert phenotypes, indicating that m^6^A regulator-related genes play crucial roles in TME modulation. To quantify the m^6^A regulator-related patterns in individual patients, we have developed a corresponding scoring system, which can be utilized for clinical treatment decisions and prognostic assessment for patients with GC.

## Results

### Identification of m6A regulator-related genes in the Meta-cohort and TCGA dataset of GC

**Figure 1:**
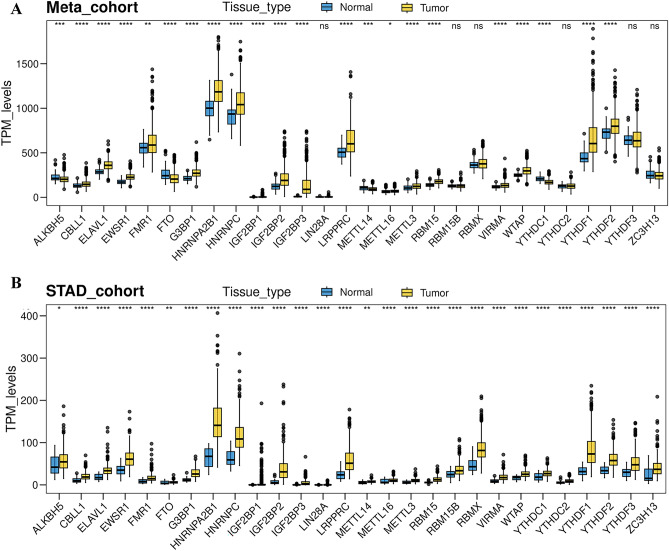
Expression of 28 m6A regulators in normal and gastric cancer (GC) samples in the Meta-cohort (**A**) and TCGA-STAD cohort (**B**).

To evaluate the potential role of m^6^A regulator-related genes, four public GC datasets from GEO were enrolled into one Meta-cohort, which consisted of 697 samples after batch effects and PCA outliers were removed (Additional files 1 and 2: Table S1 and Fig. S1A). Then, the expression data of 28 m^6^A regulators from the Meta-cohort were gathered, and their expression levels in normal and GC tissues were explored. The results showed that most of the m^6^A regulators exhibited differential expression profiles between the normal and GC samples in the Meta-cohort (Fig. 1A), and similar results were acquired when we analyzed the TCGA-STAD cohort (Fig. 1B). Subsequently, the protein expression levels of all 28 m^6^A regulators were further investigated using proteomics data from the gastric cancer (PDC000214) cohort within CPTAC project. Our findings indicated that 27 of these regulators were detected at the protein expression level. Notably, the majority of these m^6^A regulators exhibited different protein expression levels when compared to the control group (Fig. S1B and Table S2). These results showed the differential expression spectrum of m^6^A regulators, suggesting the aberrant expression of m^6^A regulators played an important role in the tumorigenesis and progression of GC.

**Figure 2:**
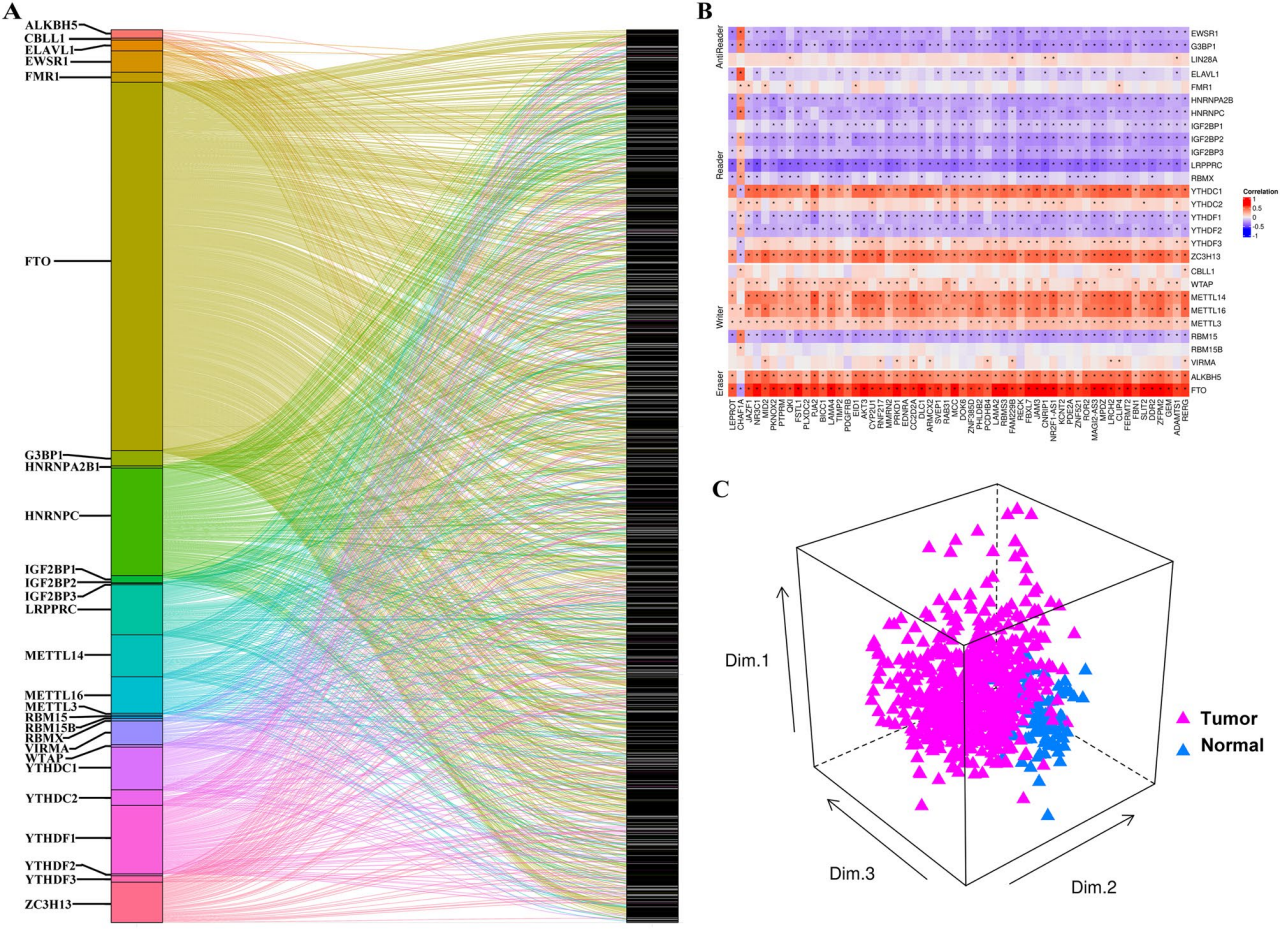
Identification of m^6^A regulator-related genes. (**A**) Sankey diagram was constructed using the ggalluvial R package to illustrate the relationships between m^6^A regulators and their related genes. (**B**) Correlation diagram was plotted to exhibit the relationship between m^6^A regulators and 56 m^6^A regulator-related genes in the Meta-cohort and presented using a heatmap via the pheatmap R package. (**C**) PCA for the expression profile of 56 m^6^A regulator-related genes to distinguish tumors from normal samples in the Meta-cohort. Two subgroups had the ability to distinguish tumors from normal samples according to the expression of 56 m^6^A regulator-related genes.

To investigate m^6^A regulator-related genes, firstly, a total of 1025 candidate genes, whose expression levels closely correlated with m^6^A regulators, were selected based on the criterion of Pearson R > 0.5; *P*-value < 0.001 (Additional file 1: Tables S3 and S4). The results demonstrated that 27 of the 28 m^6^A regulators had correlated genes, and *FTO* exhibited the largest number of related genes (505 genes), followed by *HNRNPC* (160 genes). A Sankey diagram was used to visualize the connection between m^6^A regulators and their related genes (Fig. 2A). Subsequently, univariate Cox regression analysis was performed. 56 of 1025 candidate genes found to be significantly related to the prognosis of GC in both Meta-cohort and TCGA-STAD cohort, and these were designated as m^6^A regulator-related genes (Additional files 1 and 2: Fig. S2A, Tables S5 and S6). The detailed correlation between the 56 m^6^A regulator-related genes and 28 m^6^A regulators in the Meta-cohort was illustrated in Fig. 2B. Based on the expression levels of these 56 m^6^A regulator-related genes, the GC samples and normal samples could be completely distinguished (Fig. 2C). Further validation analysis confirmed that these m^6^A regulator-related genes exhibited significantly expression profiles at both the transcriptional (GSE54129) and protein levels (PDC000214) between normal and GC samples (Fig. S2B and C and Table S7). Collectively, our study identified 56 m^6^A regulator-related genes that were closely associated with GC, and further investigating their roles in GC could provide novel insight into the pathogenesis, clinical diagnosis and prognosis of GC.

### Distinct m6A regulator-related patterns mediated by m6A regulator-related genes and hallmark gene analysis

**Figure 3:**
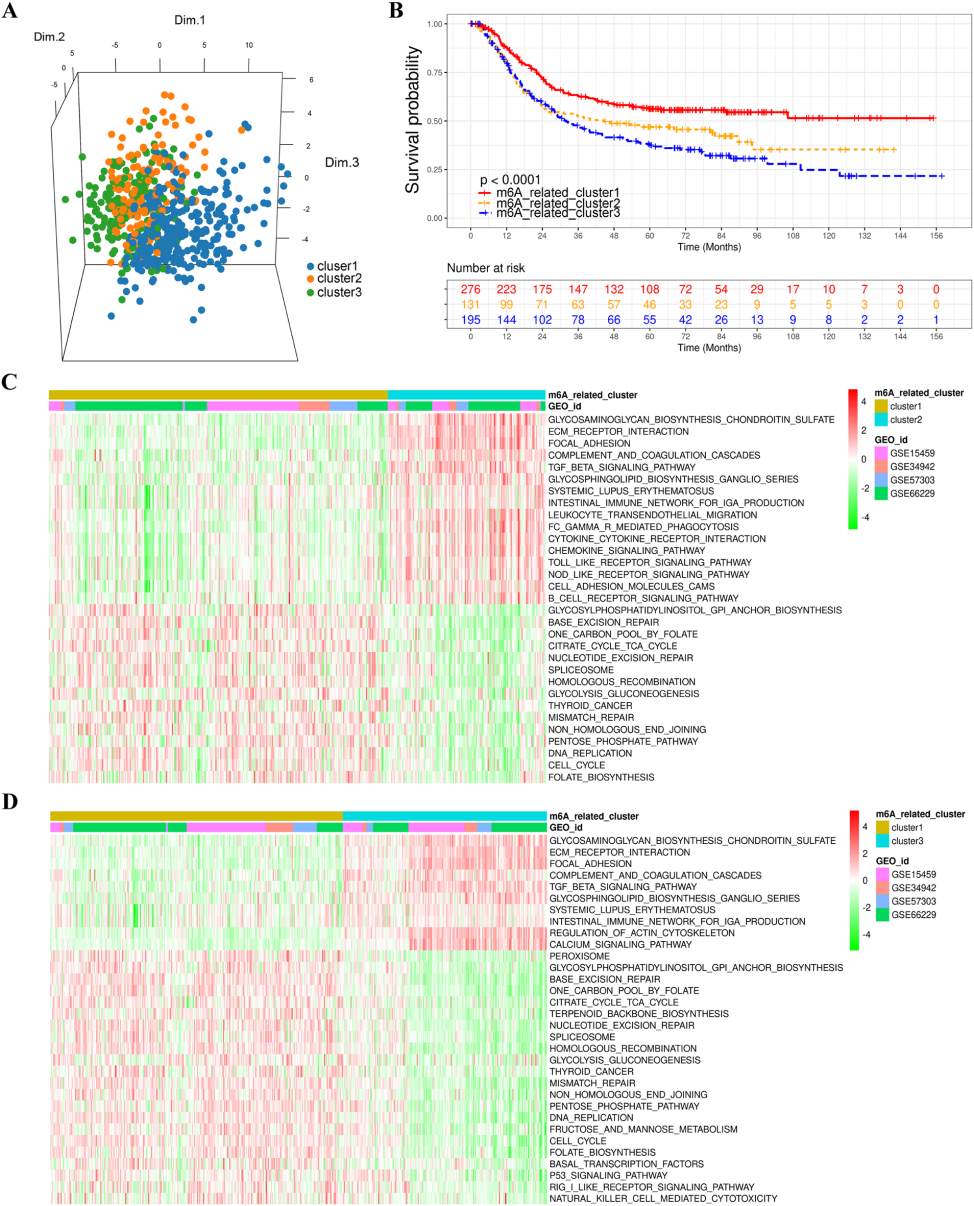
Biological features in distinct m^6^A-related patterns. (**A**) PCA for the expression profiles of three m^6^A-related patterns, indicating a significant distinction among distinct m^6^A-related patterns. (**B**) Survival analysis for the three m^6^A-related patterns in the Meta-cohort (GSE15459, GSE34942, GSE57303, and GSE66229). (**C** and **D**) GSVA enrichment analysis indicated the activation states of biological pathways in different m^6^A-related patterns. These biological processes were visualized with a heatmap. Red represents the activation pathways, and green represents the inhibited pathways.

To investigate the functions of m^6^A regulator-related genes in GC, unsupervised clustering with the R package ConsensusClusterPlus was performed to categorize patients into distinct patterns based on the expression levels of 56 m^6^A regulator-related genes. Three distinct m^6^A regulator-related patterns were eventually identified, and were abbreviated as m^6^A-related clusters 1–3 (or m^6^A-related patterns 1–3) in the following manuscript. There was a significantly different expression profiles among these three m^6^A-related clusters (Fig. 3A). Notably, m^6^A-related cluster 1 was characterized by the expression of genes, such as *CHAF1A*, *EID1*, and *MID2*, which are involved in immune activation, cell cycle regulation, cell proliferation, and DNA repair (Additional file 2: Fig. S3A). By contrast, m^6^A-related cluster 3 exhibited high expression levels of genes, such as *LRCH2*, *SLIT2*, and *DDR2*, which are primarily associated with cell-extracellular matrix interaction, angiogenesis, cytoskeleton, and cell migration (Additional file 2: Fig. S3A). Intriguingly, the majority of m^6^A regulator-related genes correlated with angiogenesis, EMT, signal transduction, and immune activation were highly expressed in m^6^A-related cluster 2 (Additional file 2: Fig. S3A). Subsequently survival analysis revealed that patients in m^6^A-related cluster 1 had a significantly better prognosis than those in m^6^A-related clusters 2 and 3 (HR, 1.48 [1.11–1.99]; Fig. 3B), which may be attributed to the distinct profiles of m^6^A regulator-related genes. These results indicated that m^6^A regulator-related genes were closely related to immune response, and investigating their roles may provide new ideas for understanding the TME modulation in GC.

### Biological annotation and TME immune infiltration in diverse m6A-related patterns

To comprehensively evaluate the biological features of m^6^A-related clusters 1–3 in TME, GSVA enrichment analysis was performed on m^6^A-related patterns of individual patients with GC. The results showed that m^6^A-related cluster 1 was significantly enriched in immune and DNA repair pathways, including natural killer cell-mediated cytotoxicity, mismatch repair, and base excision repair (Fig. 3C–D and Additional file 1: Table S8), whereas m^6^A-related cluster 3 was enriched in stromal and carcinogenic activation pathways, such as the TGF-β signal pathway (Fig. 3D and Additional file 1: Table S8). Meanwhile, m^6^A-related cluster 2 exhibited significant enrichment in stromal and immune activation pathways, including the TGF-β signal pathway, ECM receptor interaction, focal adhesion, cell adhesion molecules, and cytokine-cytokine receptor signaling pathways (Fig. 3C and Additional file 1: Table S8).

**Figure 4:**
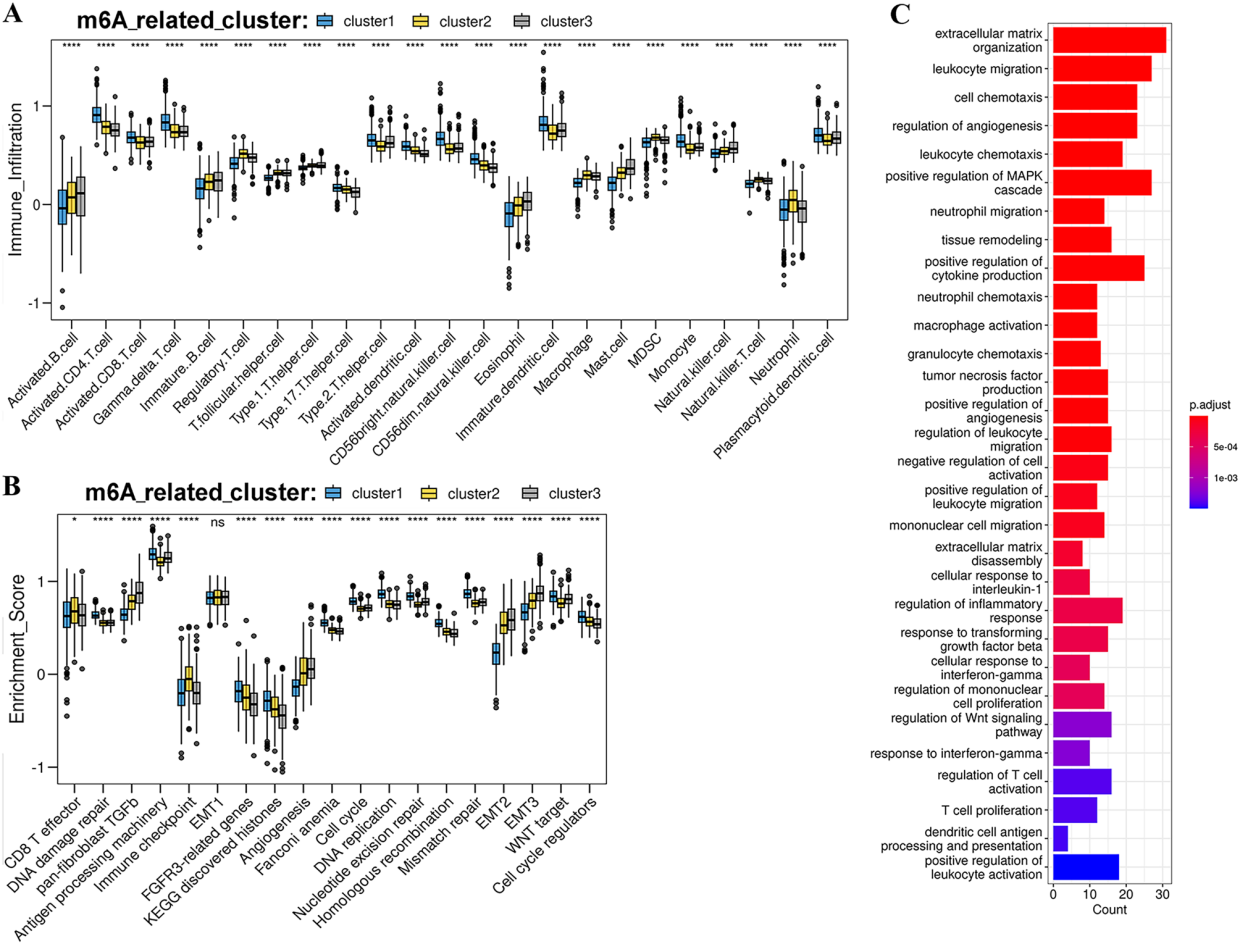
Characteristics of tumor microenvironment (TME) immune infiltration in different m^6^A-related patterns. (**A**) The relative abundance of TME cell infiltration in the three m^6^A-related patterns. The interquartile range of values was represented by the upper and lower ends of the boxes. The median values are represented by the median lines of the boxes, and the outliers are represented by the black dots. The statistical *P*-value is represented by the asterisks (*P < 0.05; **P < 0.01; ***P < 0.001). (**B**) Distinctions in innate immune-activated pathways and stromal-activated pathways among three distinct m^6^A-related patterns. The statistical differences were tested by the one-way ANOVA among the three m^6^A-related patterns. The statistical *P*-value was represented by the asterisks (*P < 0.05; ** *P* < 0.01; *** *P* < 0.001). (**C**) The functional annotation of differently expression genes (DEGs) of m^6^A regulator-related genes by GO enrichment analysis. The abundance of genes that were enriched was exhibited by the color depth of the bar plots.

To further confirm the TME landscape, we conducted immune cell infiltration analysis in these three patterns (Additional file 1: Tables S9 and S10). We found that these different m^6^A-related patterns exhibited distinct characteristics of TME immune cell infiltration (Fig. 4A). m^6^A-related cluster 1 displayed a dramatic advantage in immune activation. Most types of immune cells, including CD8^+^ T and CD4^+^ T cells, which represent immune activation, were significantly enriched in m^6^A-related cluster 1 (Fig. 4A and Additional file 1: Table S10). m^6^A-related cluster 3 exhibited the worst performance in T cell infiltration and was notably enriched in immunosuppressive cells, such as Tregs and MDSCs (Fig. 4A). Additionally, m^6^A-related cluster 2 was enriched not only in T cells but also in immunosuppressive cells (Fig. 4A). These results were consistent with the above showing that patients in m^6^A-related clusters 2 and 3 had worse survival outcomes than those in m^6^A-related cluster 1 (Fig. 3B). In addition, pathway enrichment analysis demonstrated that m^6^A-related cluster 1 was significantly enriched in the immune activation pathway, especially various pathways related to DNA repair, such as mismatch repair, the roots of microsatellite instability (MSI; Fig. 4B). Previous studies demonstrated that patients with MSI-H showed a high level of immune activation and enhanced response to immunotherapy^35,36^. These results indicated that patients in m^6^A-related cluster 1 may benefit from immunotherapy. By contrast, m^6^A-related cluster 3 was markedly enriched in the stromal activation pathways, including EMT, the TGF-β pathway, and angiogenesis (Fig. 4B), which were related to the immunosuppression of TME^37^. Notably, m^6^A-related cluster 2 was enriched in pathways related to stromal and immune activation (Fig. 4B). Based on above results, we found three m^6^A-related patterns exhibited distinct immune-infiltration profiles. m^6^A-related cluster 1 was categorized as an immune-inflamed phenotype, featured by a high level of immune cell infiltration. Similarly, m^6^A-related cluster 2 was categorized as an immune-excluded phenotype, featured by immune cell enrichment and stromal activation. Additionally, m^6^A-related cluster 3 was categorized as an immune-desert phenotype, featured by suppressed immunity. To further validate the biological characteristics in distinct m^6^A-related patterns, DEGs between these m^6^A-related patterns were identified using the “limma” R package. Then, GO enrichment analysis for 401 DEGs was conducted by the ClusterProfiler package and the enriched biological processes were listed in Additional file 1: Table S11. Results revealed that these genes were significantly enriched in biological processes related to immunity and EMT, such as extracellular matrix organization, regulation of angiogenesis, regulation of T cell activation and T cell proliferation, which reaffirmed that m^6^A -related patterns were closely related to immune modulation in TME (Fig. 4C). The above analyses demonstrated that various m^6^A-related patterns had a distinct TME cell infiltration landscape, and evaluating immune characteristics of m^6^A-related patterns will provide important insights into the understanding of TME.

### Identification of m6A-related patterns and integrated analysis of clinical traits in the ACRG cohort

**Figure 5:**
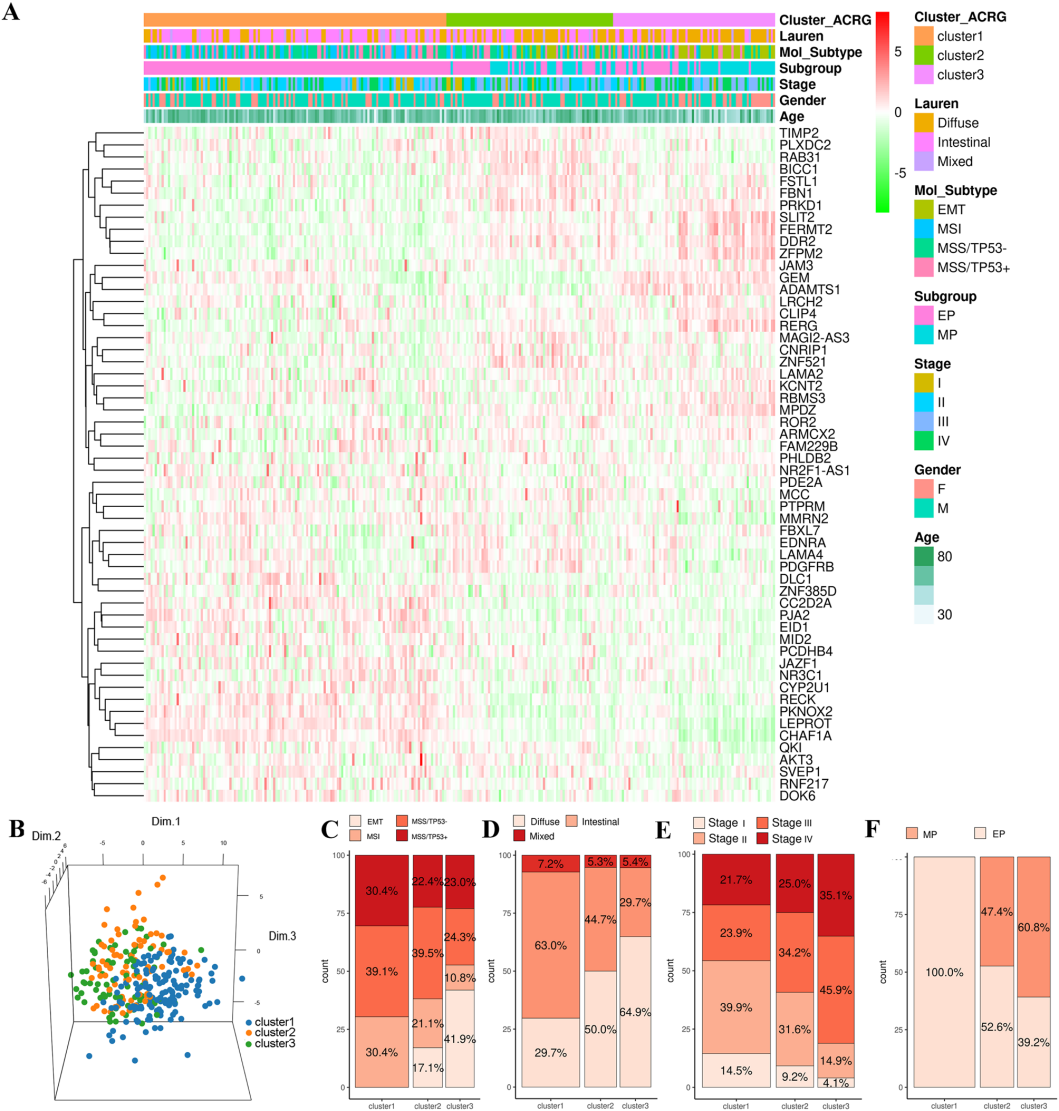
Transcript and clinical traits of different m^6^A-related patterns. (**A**) Unsupervised clustering of 56 m^6^A regulator-related genes in the ACRG cohort. The m^6^A-related patterns, the Lauren subgroups, the molecular subtypes, the subgroups, the clinical stage, gender, and age were applied to patient annotations. (**B**) PCA for the expression profiles of three m^6^A-related patterns in the ACRG cohort, indicating a significant distinction among distinct m^6^A-related patterns. (**C**–**F**) The proportion of ACRG molecular subtypes (**C**), Lauren subgroups (**D**), clinical stages (**E**), and subgroups (**F**) in three different m^6^A-related patterns.

To further investigate the biological behavior and clinical traits of these patterns, the ACRG cohort was selected as it contained the most comprehensive clinical information. Similar to the Meta-cohort clustering, unsupervised analysis also revealed three distinct m^6^A-related patterns (Fig. 5A), and significant differences in transcriptional profile were observed among these patterns (Fig. 5B, Additional file 2: Fig. S4A). m^6^A**-**related cluster 1 exhibited high expression of *CHAF1A* and a decline in the expression levels of other m^6^A regulator-related genes (Additional file 2: Fig. S4A). Meanwhile, m^6^A**-**related cluster 2 dominantly expressed *TIMP2* and *RAB31*, while m^6^A**-**related cluster 3 showed high expression of *RECK*, *FERMT2* and *SLIT2*, etc. (Additional file 2: Fig. S4A). Then, the correlation of the clinical characteristics with m^6^A-related patterns were further explored (Fig. 5C–F and Additional file1: Table S12). Patients with MSI types were characterized by m^6^A-related cluster 1, whereas patients with EMT types were characterized by m^6^A-related cluster 3 (Fig. 5C). We also noted that patients in m^6^A-related clusters 2 and 3 exhibited higher pathological stages (stages III/IV), and were enriched in the mesenchymal phenotype (MP) and diffuse histological type (Fig. 5D–F). m^6^A-related cluster 1, which was mostly enriched in the intestinal histological type and epithelial phenotype (EP), exhibited an earlier stage (stages I/II) (Fig. 5D–F). In GC, EMT type, higher pathological stages, diffuse histological type and MP were significantly related to poorer prognosis, while patients with MSI type, earlier stage, intestinal histological type and EP were associated with better clinical outcomes^38–40^. Consistent with the above results, survival analysis confirmed that m^6^A-related cluster 1 was significantly linked to prolonged survival (HR, 1.63 [1.10–2.44]), while m^6^A-related clusters 2 and 3 were related to poor survival, especially m^6^A-related cluster 3 (Additional file 2: Fig. S4B). These results revealed distinct m^6^A-related patterns exhibited different clinical characteristics and clinical prognosis. Patients with EMT types were mostly enriched in m^6^A-related cluster 3, while few were clustered into m^6^A-related cluster 1, which again reinforced m^6^A-related pattern 1 was related to immune activation and m^6^A-related pattern 3 was linked to stromal activation.

### Landscape of the immune/stromal activation-related index of m6A-related patterns

To elucidate the mechanisms of distinct m^6^A-related patterns on TME modulation, we performed a comprehensive analysis of cytokine/chemokine and immune checkpoint expression in the three m^6^A-related patterns. This approach enabled us to comprehensively evaluate the intricate interplay between m^6^A-related patterns and the immune landscape within the TME. The factors were selected according to previously published literature, of which *TGFB1*, *TGFB2*, *TGFB3*, *IL10*, *CCL22*, *PRF1*, *GZMA*, *GZMB*, *IL2*, *IL23A*, *TNF*, *IL1B*, and *CCL28* were considered to be involved in immune regulation. *CTLA4*, *IDO1*, *LAG3*, *PDCD1*, *CD274*, *PDCD1LG2*, *TIGIT*, *TNFRSF9*, *LMTK3*, and *HAVCR2* were considered as immune checkpoints. *ACTA2*, *COL4A1*, *PDGFRA*, *SMAD9*, *TGFB2*, *TGFBR2*, *CLDN3*, *ZEB1-AS1*, *VIM*, and *TWIST1* were considered to be associated with stromal activation^41–43^. Our analysis revealed that the transcripts facilitating immune activation, such as *IL23A*, *TNF*, and *IL1*, were notably upregulated in m^6^A-related clusters 1 and 2 (Additional file 2: Fig. S5A). These transcripts enhance the activation and expansion of T cells^44^. In addition, m^6^A-related cluster 2 also showed significantly evaluated expressions of transcripts related to immunosuppressive molecules, immune checkpoints, and stromal activation, especially *TGFB1* and *IL10* (Additional file 2: Fig. S5A-C). m^6^A-related cluster 3 upregulated the expression of mRNAs correlated with immunosuppressive molecules and stromal activation (Additional file 2: Fig. S5A and C), but had the lowest expression levels of transcripts related to immune activation (Additional file 2: Fig. S5A). Previous studies have demonstrated that *TGF-β*, *IL10*, and some immune checkpoints were closely correlated with the differentiation of Tregs^45,46^. Therefore, these results reaffirm our results in Fig. 4B, showing that Tregs were mainly enriched in m^6^A-related clusters 2 and 3. The aforementioned findings indicate that m^6^A-related cluster 1 is characterized by T cell activation, corresponding to the immune-inflamed phenotype; m^6^A-related cluster 2 can recruit T cells but expresses various immunosuppressive molecules and stromal activation molecules, preventing T cells from entering the tumor parenchyma, corresponding to the immune-excluded phenotype; m^6^A-related cluster 3 is featured by immune suppression, corresponding to the immune-desert phenotype.

**Figure 6:**
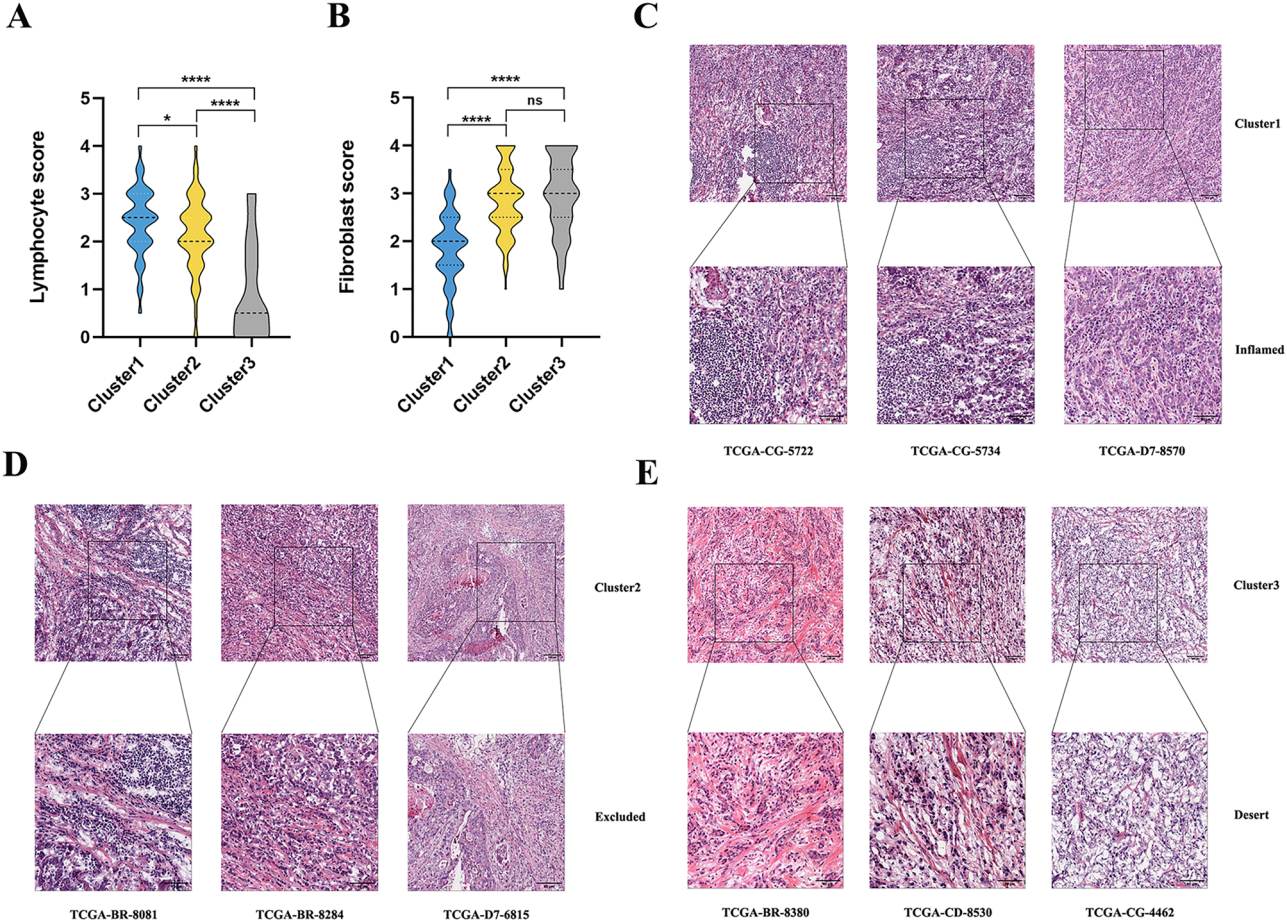
Histologic features of distinct m^6^A-related patterns. (**A**) Violin Diagram for lymphocyte scores of three different m^6^A-related patterns (n = 325); Lymphocyte was scored from 0–4 calculated by manual examining H&E staining slides. (**B**) Violin Diagram for stroma scores of three different m^6^A regulator-related patterns (n = 325); the stroma was scored from 0 to 4, calculated by manually examining the percentage of fibroblasts in H&E staining slides. (**C**–**E**) Representative TCGA H&E histology images of three different m^6^A regulator-related patterns and their correlation with distinct TME immune phenotypes: immune-inflamed (**C**), immune-excluded (**D**), immune-desert (**E**). Scale bar: 50 μm/100 μm. Statistical *P*-values are indicated by asterisk (*P < 0.05; **P < 0.01; ***P < 0.001).

Next, we conducted a profound analysis of representative GC tissue slides derived from the TCGA-STAD dataset (n = 325), with the objective of meticulously examining the phenotypic traits associated with distinct m^6^A-related patterns histologically. Through semi-quantitative pathological evaluation, we observed a significant preponderance of lymphocyte score in m^6^A-related cluster 1, demonstrating a notably higher degree of lymphocyte infiltration compared to other clusters (Fig. 6A). Subsequently, m^6^A-related cluster 2 exhibited a certain degree of lymphocyte infiltration, albeit slightly inferior to m^6^A-related cluster 1 in terms of lymphocyte score (Fig. 6A). Conversely, m^6^A-related cluster 3 displayed a significantly lower level of lymphocyte infiltration compared to m^6^A-related cluster 1 (Fig. 6A). Additionally, m^6^A-related cluster 1 exhibited the lowest stroma score (Fig. 6B). Furthermore, our analysis revealed significant disparities in the histologic characteristics of lymphocytes and stromal spatial distribution among the various m^6^A-related patterns (Fig. 6C–E). Notably, m^6^A-related cluster 1 displayed a substantial number of lymphocytes tightly infiltrating the tumor cells, accompanied by relatively low stromal infiltration (Fig. 6C). This distribution pattern mirrors the immune-inflamed phenotype, suggesting a more robust immune response^47,48^. In contrast, m^6^A-related cluster 2 exhibited a coexistence of lymphocytes and stromal infiltration, with lymphocytes primarily localized within the stroma and a comparably lower number surrounding the tumor cells (Fig. 6D). This distribution pattern is reminiscent of the immune-excluded phenotype, indicating immune activation capacity that is nevertheless constrained by stromal infiltration^47,48^. In contrast, m^6^A-related cluster 3 exhibited an extremely low lymphocyte content accompanied by stromal infiltration, which aligns with an immune-desert phenotype^47,48^ (Fig. 6E). This observation suggests a limited immune response in this cluster. These findings further corroborate the close correlation between distinct m^6^A-related patterns and varying immune phenotypes. Consequently, identification of m^6^A-related patterns offers a valuable approach for analyzing the TME immune phenotypes of GC patients, providing significant insights into the prognosis and immunotherapy responsiveness of these individuals.

### Construction of m6A-related signatures and characterization of clinical and biological traits

**Figure 7:**
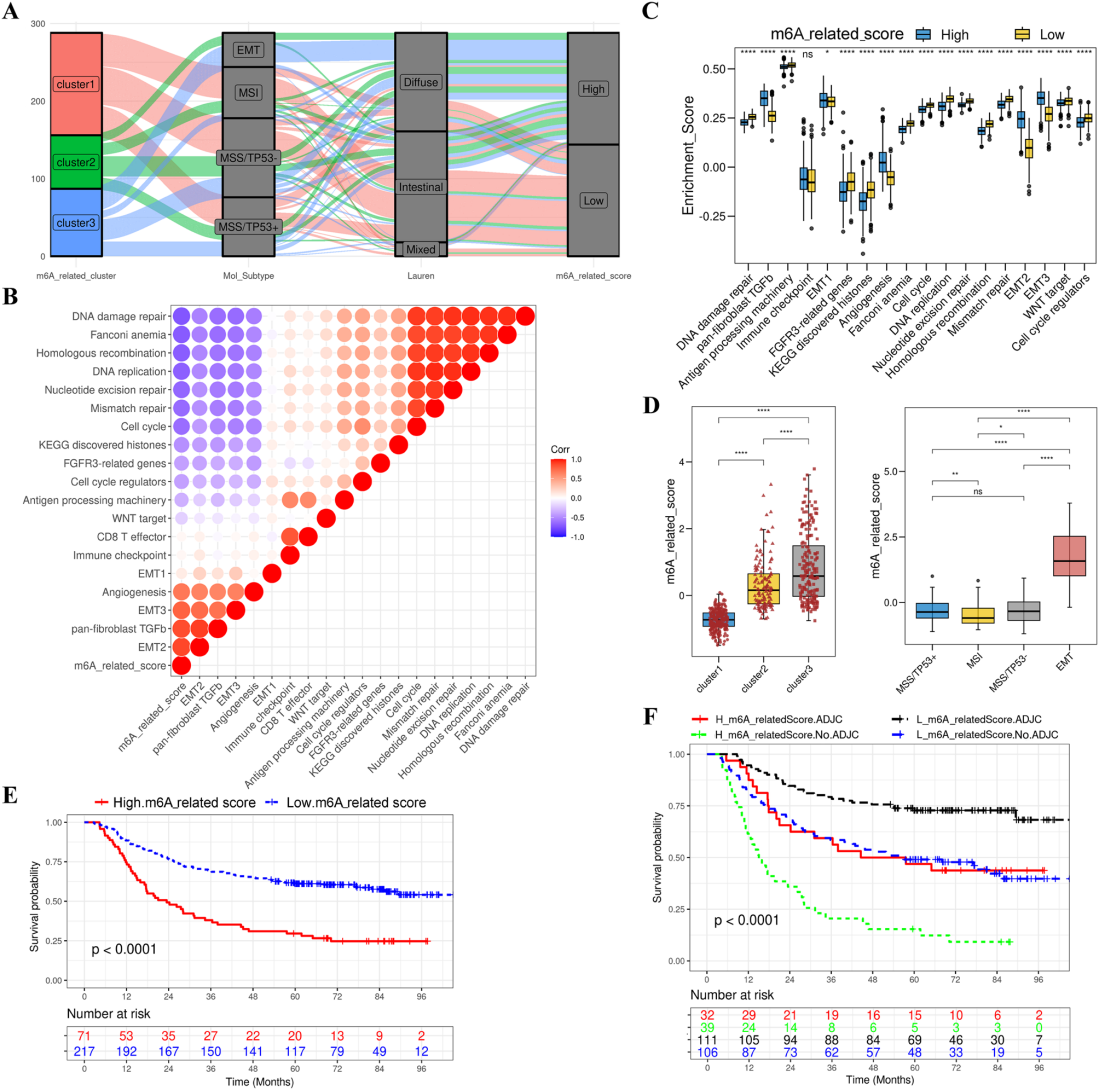
Establishment of m^6^A signatures. (**A**) Dynamic changes in the m^6^A-related patterns, ACRG molecular subtypes, and m^6^A-related scores are illustrated by an alluvial diagram. (**B**) The relationship between m^6^A-related scores and the known gene signatures in the ACRG cohort by Spearman analysis. Blue represents the negative correlation, and red represents the positive correlation. (**C**) Distinction in innate immune-activated pathways and stromal-activated pathways between m^6^A-related scores. Statistical differences were tested by one-way ANOVA between m^6^A-related scores. The statistical *P*-value was represented by the asterisks (*P < 0.05; **P < 0.01; ***P < 0.001). (**D**) Differences in m^6^A-related scores among three different m^6^A-related patterns and among four ACRG molecular subtypes. (**E**) Survival analysis for high (71 cases) and low (217 cases) m^6^A-related score groups in the ACRG cohort by Kaplan–Meier curves (HR, 1.63 [1.10–2.44]); *P* < 0.0001, Log-rank text). (**F**) Survival analysis was used for patent groups classified into four groups based on m^6^A-related scores and treatment with adjuvant chemotherapy using Kaplan–Meier curves. H, high; L, low; ADJC, adjuvant chemotherapy (*P* < 0.0001, Log-rank test).

The m^6^A-related patterns play a crucial role in shaping distinct TME immune-infiltration phenotypes. However, we cannot use m^6^A-related patterns to predict TME characteristics in individual patients, as the aforementioned analyses primarily focused on patient populations. Given the complexity of m^6^A-related patterns and individual heterogeneity, we devised a scoring system to quantify these patterns in individual patients according to the 56 m^6^A regulator-related genes. We designated this scoring system as the m^6^A-related score. First, the properties of individual patients were investigated and visualized using an alluvial diagram (Fig. 7A), which showed that patients in m^6^A-related cluster 1 had a lower m^6^A-related score than those in m^6^A-related cluster 3. To further evaluate the significance of m^6^A-related score, we examined the relationship between m^6^A-related scores and some well-known signatures. The m^6^A-related score was positively correlated with stromal activation-related signatures, but negatively with DNA repair-related signatures (Fig. 7B and Additional file 1: Table S13). The analyses for immune activation and stromal activation showed low scores were significantly associated with DNA damage repair and mismatch repair, whereas high scores were related to EMT, TGF-β pathway, and angiogenesis (Fig. 7C). Similarly, Kruskal–Wallis tests found a significant difference of m^6^A-related score between different m^6^A-related patterns. m^6^A-related cluster 1 exhibited the lowest median m^6^A-related score, whereas m^6^A-related cluster 3 had the highest score (Fig. 7D). In addition, patients with EMT types exhibited the highest m^6^A-related score compared to other ACRG types (Fig. 7D). These results demonstrate that a high m^6^A-related score is linked to stromal activation, while a low m^6^A-related score is associated with immune activation.

Subsequently, we further evaluated the value of the m^6^A-related score in predicting prognosis for patients with GC. At the cutoff value 0.34 according to the survminer package, patients were categorized into high or low m^6^A-related score groups. Survival analysis demonstrated that the high m^6^A-related score group had poor prognosis (Fig. 7E). Whether the m^6^A-related scores could serve as independent prognostic biomarkers for patients with GC was also investigated. A multivariate Cox regression model, considering age, gender, stage, and TNM status of patients, confirmed m^6^A-related scores were robust and independent prognostic biomarkers for patients with GC (HR, 1.78 [1.44–2.20] in the Meta-cohort; Additional file 2: Fig. S6A; HR, 1.38 [1.15–1.66] in the TCGA-STAD cohort; Additional file 2: Fig. S6B). We tested the performance of the m^6^A-related score in predicting the efficacy of adjuvant chemotherapy in patients with GC. The results demonstrated that patients with low m^6^A-related scores and receiving adjuvant chemotherapy had significantly high survival rates (HR, 2.70 [1.26–5.80]; Fig. 7F). Furthermore, patients with low m^6^A-related scores exhibited high survival rates with or without receiving adjuvant chemotherapy (Fig. 7F). Finally, we demonstrated that patients with diffuse histological subtype, elder age, advanced stage, and EMT type are notably related to a higher m^6^A-related score, which suggested these patients were corresponding to m^6^A-related cluster 3 and immune-desert phenotype, with poor prognosis (Additional file 2: Fig. S6C). These results demonstrated the m^6^A-related score was an independent and robust prognosis biomarker for patients with GC and can be used to evaluate clinical characteristics.

### Characteristics of m6A-related scores in TCGA-STAD cohort and tumor somatic mutation analysis

**Figure 8:**
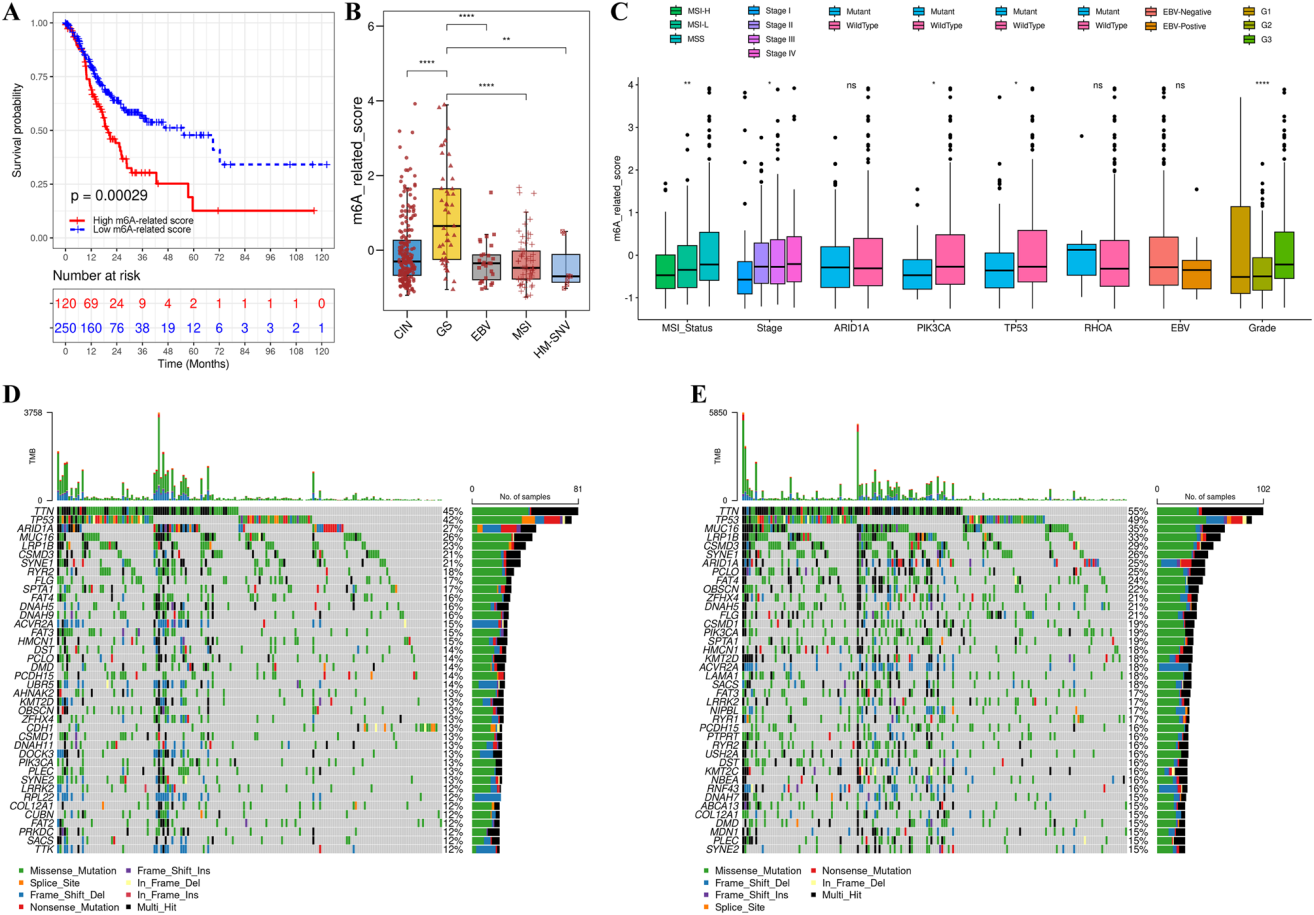
Characteristics of m^6^A modification in the TCGA subtypes and tumor somatic mutation. (**A**) Survival analysis for high (120 cases) and low (250 cases) m^6^A-related score groups in the TCAG cohort by Kaplan–Meier curves (HR, 1.82 [1.27–2.62]); *P* < 0.0001, Log-rank text). (**B**) Variance in m^6^A-related scores among various TCGA-STAD molecular subtypes. The interquartile range of values was represented by the upper and lower ends of the boxes. The median values were represented by the lines in the boxes. The statistical *P*-values were represented by the asterisks (*P < 0.05; **P < 0.01; ***P < 0.001). **(C)** Variance in m^6^A-related scores among different MSI statuses, clinical stages, EBV infections, and clinical grades. The interquartile ranges of the values are represented by the upper and lower ends of the boxes. The median values are represented by the lines in the boxes. The statistical *P*-values are represented by the asterisks (*P < 0.05; **P < 0.01; ***P < 0.001). (**D** and **E**) The tumor somatic mutation of high scores (**D**) and low scores (**E**) is visualized by a waterfall plot. Each column represented an individual patient. The upper bar plot represents TMB, and the right numbers represent the mutation frequency.

A comprehensive molecular phenotype has been built for GC by TCGA project, which categorized patients with GC into five molecular subtypes, including chromosomal instability, genome stability (GS), EBV infection, MSI, and hypermutated single nucleotide variation (HM-SNV)^49,50^. We evaluated the distinction m^6^A-related score between these molecular subtypes. Our results revealed that the high m^6^A-related score group was significantly enriched in the GS subtype and exhibited a poor prognosis with a median survival time of approximately 5.08 years, whereas the low m^6^A-related score group was focused on the molecular subtypes of HM-SNV, MSI, and EBV, which were linked to survival benefit (median survival time of approximately 6.92 years; Fig. 8A and B). Multivariate Cox regression analysis verified that the m^6^A-related score is an independent prognostic biomarker for patients with GC (Additional file 2: Fig. S6B). Moreover, elevated m^6^A-related scores were observed predominantly in the MSS molecular subtype, advanced stage patients, and G3 patients, which were associated with poor prognosis (Fig. 8C). Previous studies have showed that the molecular subtypes of GC are closely related to clinical response to immunotherapy. GC patients with the EBV-positive, MSI and HM-SNV molecular subtypes exhibited improved efficacy after immunotherapy, as the increasing expression of PD-L1 or a high tumor mutational burden (TMB)^49,51,52^. In our study, patients with MSI and EBV molecular subtypes were significantly related to m^6^A-related cluster 1 with low m^6^A-related score, whereas patients with GS molecular subtype were related to m^6^A-related cluster 3 with a high m^6^A-related score (Additional file 2: Fig. S7A). Similar analysis revealed that patients with high differentiation (G III), early stage (stage I), and MSI molecular subtype were concentrated in m^6^A-related cluster 1 (Additional file 2: Figs. S7A–D).

Clinical trials have shown that tumor somatic mutations are closely linked to immunotherapy. Higher somatic mutation rates led to an efficient response to ICIs, such as anti-PD-1/PD-L1 immunotherapy^53^. Then, the differences in somatic mutations between high and low m^6^A-related score groups were investigated in the TCGA-STAD cohort. We found high m^6^A-related score group exhibited lower TMB status than low m^6^A-related score group, with the rate of the 40^th^ highest mutated genes being 12% versus 15% (Fig. 8D–E). This observation was verified using TMB quantification analysis, as the high m^6^A-related score group had a lower TMB score (Additional file 2: Fig. S7E). Similarly, correlation analysis demonstrated that the m^6^A-related score was negatively correlated with the TMB status (Additional file 2: Fig. S7F). Accumulated results have reported that patients with high TMB status showed a better response to immunotherapy^54^. Therefore, the above results indicated that the distinct m^6^A-related patterns may be an important factor for the response to immunotherapy, and evaluating the m^6^A-related score of individual patients may be beneficial for predicting efficacy of immunotherapy.

Previous investigations have demonstrated that patients with higher TMB status exhibited a better response to ICIs. The mutation of some crucial genes may have a close relationship with sensitivity or resistance to immunotherapy. For genes in TCGA-STAD cohort such as *TP53* and *PIK3CA*, wild type has remarkably higher m^6^A-related score compared to mutant type, whereas there was no difference in *RHOA* and *ARID1A* (Fig. 8C). The results above suggested exploring the mechanism of m^6^A-related patterns in TMB, which may provide new perspective for TME modulation and ICIs.

### Clinical value of m6A-related scores for predicting prognosis and immunotherapy with anti-PD-1/L1 and anti-CTLA4

Our m^6^A-related scoring system exhibited predictive value in the TCGA-STAD cohort, confirming its potential as a prognostic biomarker for GC. To further explore its prognostic efficiency, we verified it in other independent GC cohorts, including GSE15459, HR = 2.14 (1.43–3.22); GSE34942, HR = 1.52 (0.67–3.46); GSE57303, HR = 1.57 (0.80–3.09); and GSE26253, HR = 2.0 (1.43–2.82; Fig. S8A). Furthermore, we extended the m^6^A-related scoring system to all digestive system tumors, including esophageal carcinoma, colorectal adenocarcinoma, cholangiocarcinoma, pancreatic adenocarcinoma, and hepatocarcinoma in the TCGA database (HR, 1.76 [1.42–2.17]; Additional file 2: Fig. S8E) and even the combined set of all TCGA tumor types (HR, 2.58 [2.38–2.80]; Additional file 2: Fig. S8F). The above results demonstrated that m^6^A-related score exhibited an excellent prognostic value. ROC curves further confirmed the predictive performance of m^6^A-related scoring system, especially in elderly patients (Additional file 2: Figs. S8G and H).

**Figure 9:**
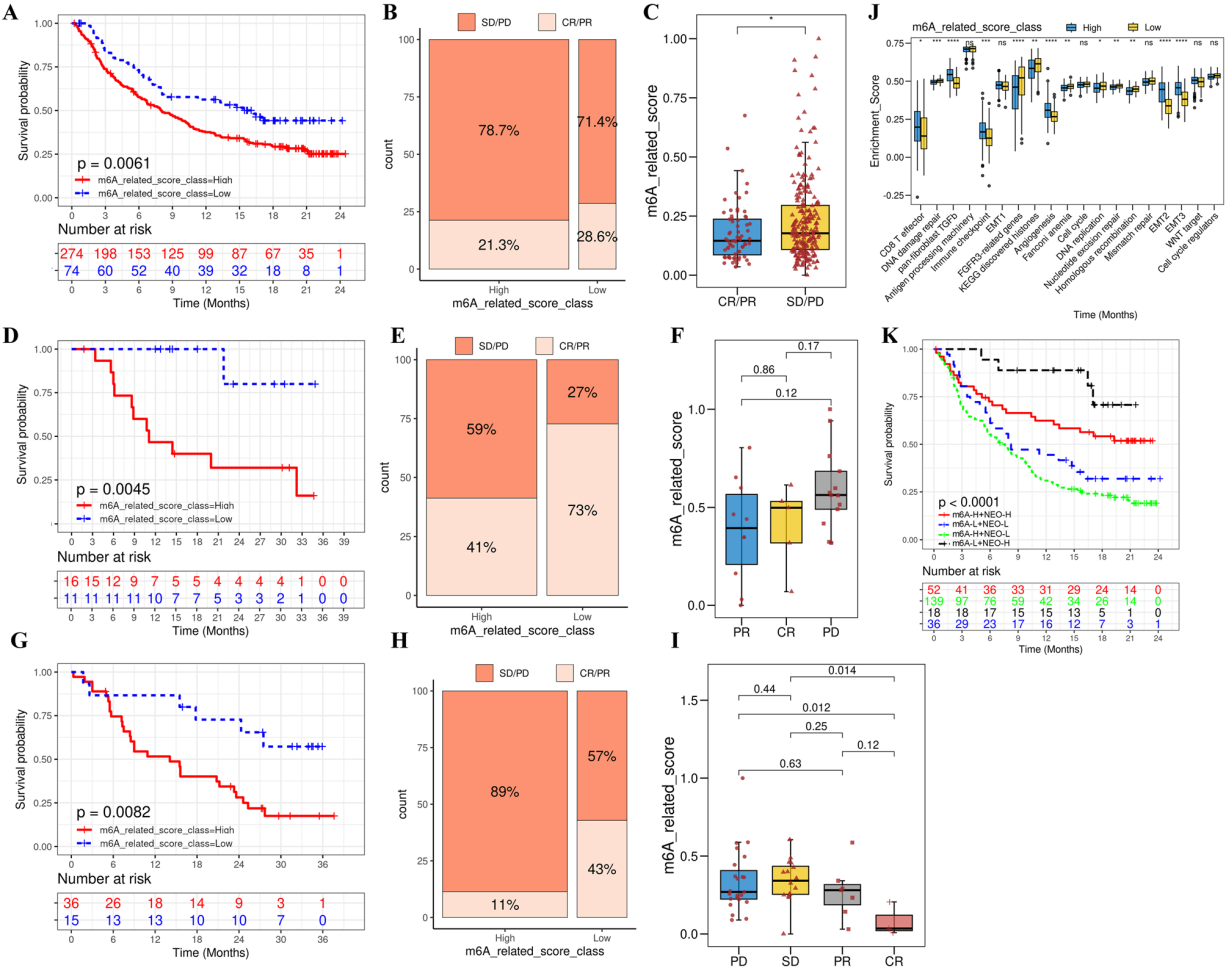
Role of the m^6^A-related scoring system in immunotherapy. (**A**–**C**) Survival analysis and clinical benefit for low (74 cases) and high (274 cases) m^6^A-related score groups in the anti-PD-L1 immunotherapy cohort (IMvigor210 cohort; HR = 1.62 [1.20–2.19]; *P* = 0.006, log-rank test). (D–F) Survival analysis and clinical benefit for low (11 cases) and high (16 cases) m^6^A-related score groups in the anti-PD-1 immunotherapy cohort (GSE78220 cohort; HR = 10.55 [3.40–32.72]; *P* = 0.005, log-rank test). (G–I) Survival analysis and clinical benefit for low (15 cases) and high (36 cases) m^6^A-related score patient groups in the anti-CTLA4 immunotherapy cohort (GSE91061 cohort; HR = 3.02 [1.52–6.01]; *P* = 0.008, log-rank test). (**J**) Differences in distinct m^6^A-related scores in TME immune cell infiltration. The interquartile ranges of the values are represented by the upper and lower ends of the boxes. The median values are represented by the lines in the boxes. The statistical *P*-values are represented by the asterisks (*P < 0.05; **P < 0.01; ***P < 0.001). (**K**) Survival analysis of anti-PD-L1 immunotherapy for both the m^6^A-related score and neoantigen burden by Kaplan–Meier curves. H, high; L, low; NEO, neoantigen burden (*P* < 0.0001, Log-rank test).

Immunotherapies including PD-1, PD-L1 and CTLA4 blockade have been recognized as a major breakthrough in GC therapy. Thus, we explored the effect of m^6^A-related score in predicting the response to ICIs in patients from three immunotherapy cohorts: the anti-PD-L1 cohort (IMvigor210), the anti-PD-1 cohort (GSE78220), and the anti-PD-1/anti-CTLA4 cohort (GSE91061). Among these three cohorts, patients with low m^6^A-related score had prolonged survival and improved response to ICIs (Fig. 9A–I; IMvigor210, HR = 1.62 [1.20–2.19], Fig. 9A; GSE78220, HR = 10.55 [3.40–32.72], Fig. 9D; GSE91061, HR = 3.02 [1.52–6.01], Fig. 9G). The response to immunotherapy and therapeutic advantage in patients with a low m^6^A-related score were verified (Fig. 9B and C; E and F and H and I). In addition, further investigations revealed Angiogenesis, WNT target and EMT were remarkable activated in patients with high m^6^A-related score, which mediated immune suppression of TME (Fig. 9J). Meanwhile, pathways related to DNA repair, such as DNA damage repair, Nucleotide excision repair, Mismatch repair and homologous recombination, were significantly activated in patients with a low m^6^A-related score, which was associated with improved response to immunotherapy. Moreover, another indicator closely related to immunotherapy, tumor neoantigen burden, was also evaluated. The results showed that patients with a low m^6^A-related score exhibited remarkable therapeutic advantages among patients with a high tumor neoantigen burden (Fig. 9K). Consequently, the above results suggested that m^6^A-related patterns had a close relationship with TME phenotypes and response to immunotherapy, and evaluating the m^6^A-related pattern/score in individual patients would improve the clinical efficacy prediction of immunotherapy.

## Discussion

Accumulating evidence has proved the m^6^A modification plays an essential role in regulating immune cell infiltration and angiogenesis in TME by interaction with m^6^A regulators^55–57^. Meanwhile, m^6^A regulator-related ncRNAs were also reported to play an indispensable role in diagnosis, prognosis and even modulating TME in various tumors^58–60^. Nevertheless, most investigators mainly focused on m^6^A regulator-related ncRNAs. The comprehensive roles of m^6^A regulator-related genes, both coding genes and ncRNAs, in TME infiltration characterization are not well recognized. An integrated analysis of the functions of m^6^A-related patterns in GC TME modulation will enhance our understanding of TME antitumor immunity, and guide immunotherapy strategies.

In this study, 56 m^6^A regulator-related genes, comprising 54 coding genes and 2 ncRNA genes, were eventually identified. Based on the expression patterns of these related genes, three distinct m^6^A-related patterns were also established. Our findings revealed these three patterns have distinct TME infiltration landscape, respectively. m^6^A-related cluster 1 was featured by immune activation, corresponding to an immune-inflamed phenotype; while m^6^A-related cluster 2 was featured by stromal and immune activation, corresponding to an immune-excluded phenotype, m^6^A-related cluster 3 was featured by immune suppressing, corresponding to an immune-desert phenotype. The immune-inflamed phenotype is characterized by abundant infiltration of immune cells, clinically referred to as “hot” tumors, which exhibit favorable responsiveness to immunotherapy^61^. In contrast, the immune-excluded phenotype and the immune-desert phenotype, known as “cold” tumors, are characterized by little immune cell infiltration in TME^61–63^. Although the immune-excluded phenotype possesses abundant of immune cells, the immune cells mainly reside in the periphery of tumors parenchyma, rather than infiltrating the TME^62,63^. In line with our investigations, m^6^A-related cluster 1 we identified exhibits significant infiltration of T cells, monocytes, and antigen-presenting cells in TME (Fig. 4A) and infiltration of lymphocytes surrounding the tumor cells, resembling an immune-inflamed phenotype (Fig. 6C). This is also accompanied by a higher level of pro-inflammatory factors, such as IL-23, TNF-α and IL-1β, which are considered to be associated with immune activation^44^. m^6^A-related cluster 2 exhibited a notable infiltration of not only a substantial number of Tregs and MDSCs but also a considerable population of CD4^+^ T cells and CD8^+^ T cells (Fig. 4A). Furthermore, this cluster expressed a high abundance of transcripts linked to stromal activation (Fig. S5C). Pathological evaluation revealed that these lymphocytes were predominantly infiltrated in the stroma (Fig. 6D), suggesting a further suppression of immune activation. Notably, these features closely resembled an immune-excluded phenotype, indicating a potential mechanism underlying the immune evasion within the TME. m^6^A-related cluster 3, characterized by high expression levels of TGF-β, IL-10, IDO1, and EMT, exhibits a state of extensive stromal activation (Fig. S5A and B), which are all regarded as immune suppression^64–67^. Notably, this cluster shows an absence of infiltrating immune activation-associated cells (Figs. 4A and 6E), indicating a suppressive effect on immune activation within the TME, corresponding to an immune-desert phenotype. Additionally, an intriguing result was found when investigating the cytokines/chemokines profiles among three distinct clusters. The m^6^A-related cluster 1 exhibits a higher proportion of CD8^+^ T cells, but a lower level of GZMB (Fig. S5A and B), which seems to be contradictory to the traditional theory that CD8^+^ T cells are the primary source of GZMB secretion^68^. Recent studies have reported that Tregs, besides CD8^+^ T cells, are the primary source of GZMB secretion in TME^69^. More importantly, Tregs could modulate the TME to immune suppressive status through releasing GZMB, which concurrently induces apoptosis of effector T cells. In our research, both Tregs and GZMB are significantly enriched in m^6^A-related clusters 2 and 3, which suggesting Tregs may be the main source of GZMB release, and also may be the crucial manipulator for modulating the TME.

With the development of immunotherapy, the efficacy of immunotherapy has been clinically proven. However, only a small percentage of patients with cancer can benefit from immunotherapy^70,71^. Currently, predictive biomarkers for immunotherapy include not only the expression of immune checkpoints but also TMB, MSI, deficient mismatch repair genes (dMMR), and infiltration of tumor-specific T cells, all of which can predict the sensitivity of patients to immunotherapy^72–74^. dMMR impairs the ability to rectify mutated genes, resulting in elevated microsatellite instability (MSI-H) and subsequently increased TMB, eventually facilitating the recruitment of immune cells and enhancing the responsiveness to immunotherapy^75,76^. In our study, mismatch repair, base repair and other related pathways were enriched in the m^6^A-related cluster 1 (Fig. 3C and D), subsequently leading to higher MSI and TMB levels (Figs. 5C and S7A). Further survival analysis also revealed that individuals in the m^6^A-related cluster 1 have a better clinical prognosis and clinical efficacy after immunotherapy (Fig. 3B). Moreover, the expression levels of some immune checkpoint genes, which were the primary targets of immunotherapy, were also analyzed. Some star genes related to immunotherapy, such as PD-1, PD-L1 and CTLA4, exhibited relatively lower levels in m^6^A-related cluster 1 (Fig. S5B), despite the fact that patients in cluster 1 obtained better clinical benefits from immunotherapy. These results suggest that the predicting the clinical benefits from immunotherapy is extremely complicated, and various factors, such as TMB, MSI, immune checkpoint genes and maybe some unknown factors, may play an important role in improving the efficacy of immunotherapy. Combining multiple factors building efficient models for immunotherapy efficacy prediction is also urgently needed. Therefore, distinct m^6^A-related patterns exhibit different responsiveness to immunotherapy, which may serve as potential predictive biomarkers for immunotherapy.

Further, considering the heterogeneity among individual patients, we developed an m^6^A-related scoring system to assess the m^6^A-related patterns of individual GC patients. The m^6^A-related cluster 1 featuring an immune-inflamed phenotype exhibited lower m^6^A-related score, while the m^6^A-related clusters 2 and 3, featuring immune-exclude and immune-desert phenotypes, showed higher m^6^A-related scores. These results indicated that m^6^A-related score could be used to comprehensively evaluate individual patients with GC, confirming the TME immune infiltration patterns which are determining factors for immunotherapy. In addition, integrated analyses of the correlation of m^6^A-related score with various clinical traits also showed that patients with EMT and GS subtypes, closely linked to immune suppressive TME and a low response to immunotherapy^77,78^, were associated with higher m^6^A-related scores. However, patients with MSI, MSS and EBV subtypes, who had an improved response to immunotherapy^79–81^, had lower m^6^A-related scores. Meanwhile, the immunotherapy cohort results confirmed lower immunotherapy responsiveness in the high m^6^A-related score group, whereas the low m^6^A-related score group primarily consisted of patients from m^6^A-related cluster 1 (Fig. 7D), showing significantly higher clinical benefits from immunotherapy (Fig. 9). GSVA analysis also revealed that m^6^A-related scores were closely associated with pathways of EMT signal and stromal activation (Fig. 9), underlining the core role of stromal activation in predicting response to immunotherapy^8,82,83^. Subsequently, the predictive value of m^6^A-related score was appraised across various cohorts and tumor types. Our findings revealed that the m^6^A-related signature exhibited exceptional efficacy in other GC cohorts, as well as in the combined cohort of all TCGA tumors, thus underscoring the potential diagnostic value for other tumors beyond GC. Nonetheless, more research is needed to completely address this issue. Consequently, the m^6^A-related score could serve as a robust potential biomarker to predict the prognosis and clinical benefits of immunotherapy for GC patients. The integrated of m^6^A-related score with other biomarkers including TMB, neoantigen and tumor subtypes, could provide more effective immunotherapeutic strategies for individual patients with GC.

In summary, our study depicts a comprehensive landscape of TME infiltration characteristics mediated by m^6^A regulator-related genes in GC. First, we explored the characteristics of distinct m^6^A-related patterns in TME, expanding the significant role of m^6^A regulator-related genes in shaping TME diversity and the clinical or biological traits of GC. Second, we constructed an m^6^A-related scoring system for individual patients with GC. This scoring system provides an independent prognostic biomarker for prognosis and clinical benefits of immunotherapy and may facilitate the development of effective immunotherapy strategies and personalized treatment plans.

## Materials and methods

### Acquisition of GC datasets and preprocessing

In this study, we acquired six publicly available GC datasets (GSE15459, GSE34942, GSE57303, GSE26253, GSE66229, GSE54129, and TCGA-STAD) from the Gene Expression Omnibus (GEO) and the Cancer Genome Atlas (TCGA) databases to conduct further investigations (Additional file 1: Table S1)^84–89^. For microarray data obtained from the Affymetrix Human Genome U133 Plus platform, we retrieved the raw “CEL” files and utilized a powerful multiarray averaging approach, employing the affy and simpleaffy packages, to perform background adjustment and quantile normalization. In the case of microarray data from alternative platforms, the normalized matrix files were obtained directly. With regard to datasets in TCGA, RNA sequencing data, measured in transcripts per million (TPM) values, were procured from the Genomic Data Commons (GDC) (https://portal.gdc.cancer.gov/). Four GEO datasets (GSE15459, GSE34942, GSE57303, GSE66229), totaling n samples = 726, from Affymetrix were enrolled into one Meta-cohort. Batch effects in our Meta-cohort from non-biological technical biases were adjusted by the “ComBat” algorithm of the sva package. Of the 726 samples in the Meta-cohort, 29 samples did not pass quality control due to being identified as PCA outliers, resulting in the final Meta-cohort of 697 samples. These datasets were processed and then harmonized as illustrated in Additional file 1 and Additional file 2: Figure S1 and Table S1. Proteomics data of m^6^A regulators and m^6^A regulator-related genes were obtained from PDC00024 (https://pdc.cancer.gov/pdc/study/PDC000214) for subsequent research^90^ and the “limma” R package was used to compare the differences in proteomics expression between normal and GC samples. The somatic mutation data utilized in this study was obtained from the TCGA database. Analysis of the data was conducted using R software (version 4.3.0) and R Bioconductor packages.

### Acquisition of GC samples

The GC samples utilized in this study were sourced from the GEO database and the TCGA database. A total of 726 GC samples were obtained from the GEO database, among which 28 samples were subsequently excluded due to the removal of PCA outliers and adjustment for batch effects. This screening process yielded 697 GC samples for further analysis. All GC samples retrieved from the TCGA database were included in our investigation. Notably, GC samples lacking survival data were excluded from survival analyses.

### Identification of m6A regulator-related gene signature

Initially, we identified 28 m^6^A methylation regulators from previously published literature, including 8 writers (*CBLL1*, *WTAP*, *VIRMA*, *RBM15B*, *RBM15*, *METTL3*, *METTL14*, and *METTL16*), 2 erasers (*ALKBH5*, *FTO*), 15 readers (*IGF2BP1*, *IGF2BP2*, *IGF2BP3*, *ELAVL1*, *FMR1*, *HNRNPA2B1*, *HNRNPC*, *LRPPRC*, *RBMX*, *ZC3H13*, *YTHDC1*, *YTHDC2*, *YTHDF1*, *YTHDF2*, *YTHDF3*), and 3 anti-readers (*EWSR1*, *G3BP1*, *LIN28A*). Subsequently, Pearson’s correlation analysis was used to screen m^6^A regulator-related genes, resulting in the acquisition of 1025 genes satisfying the criteria of |Pearson R|> 0.5 and *P* < 0.001 in both the Meta-cohort and the TCGA-STAD cohort. We used the “ggalluvial” R package to map a Sankey diagram in order to visualize the relationships between various m^6^A regulators and their related genes^91^. These genes were selected as potential candidates for further investigation. Following this, Univariate Cox regression analysis was performed on the candidate genes, and 56 genes with *P*-values less than 0.05 in both the Meta-cohort and the TCGA-STAD cohort were designated as the m^6^A regulator-related gene signature.

### Unsupervised clustering for 56 m6A regulator-related genes

We aimed to identify distinct m^6^A regulator-related patterns in GC by analyzing 56 m^6^A regulator-related genes obtained from four GEO datasets. These genes comprised *LEPROT*, *CHAF1A*, *JAZF1*, *NR3C1*, *MID2*, *PKNOX2*, *PTPRM*, *QKI*, *FSTL1*, *PLXDC2*, *PJA2*, *BICC1*, *LAMA4*, *TIMP2*, *PDGFRB*, *EID1*, *AKT3*, *CYP2U1*, *RNF217*, *MMRN2*, *PRKD1*, *EDNRA*, *CC2D2A*, *DLC1*, *ARMCX2*, *SVEP1*, *RAB31*, *MCC*, *DOK6*, *ZNF385D*, *PHLDB2*, *PCDHB4*, *LAMA2*, *RBMS3*, *FAM229B*, *RECK*, *FBXL7*, *JAM3*, *CNRIP1*, *NR2F1-AS1*, *KCNT2*, *PDE2A*, *ZNF521*, *ROR2*, *MAGI2-AS3*, *MPDZ*, *LRCH2*, *CLIP4*, *FERMT2*, *FBN1*, *SLIT2*, *DDR2*, *ZFPM2*, *GEM*, *ADAMTS1*, and *RERG*. We performed Univariate Cox regression analysis to evaluate the correlation between these genes and the prognosis of GC. Furthermore, we used unsupervised clustering analysis to classify the distinct m^6^A regulator-related patterns, based on the expression profiles of the 56 m^6^A regulator-related genes. The ConsensusClusterPlus package was utilized for this analysis^34^, and we conducted 1000 repetitions to ensure the stability of the classification.

### Functional annotation and Gene set variation analysis (GSVA)

To gain insights into the functional roles of m^6^A regulator-related genes, we performed functional annotation using the clusterProfiler R package^34^, applying a cutoff value of FDR less than 0.05. We also conducted Gene Set Variation Analysis (GSVA) enrichment analysis to investigate the differences in biological processes among various m^6^A regulator-related patterns. The GSVA enrichment analysis was performed using the “GSVA” R package^92^, and the analysis was run using gene sets of “c2.cp.kegg.v6.2.symbols” downloaded from MSigDB. We deemed adjusted *P*-values less than 0.05 to be statistically significant.

### Exploration of TME cell infiltration

To quantify the relative abundance of each cell infiltration in the TME of GC, we adopted the single-sample gene-set enrichment analysis (ssGSEA) algorithm. We obtained gene sets for various human immune cell subsets, including activated dendritic cells, activated B cells, helper T cells, activated CD4 T cells, activated CD8 T cells, regulatory T cells (Tregs), natural killer T cells, macrophages, MDSCs and others, from previous studies^93,94^. We employed these gene sets to calculate the enrichment scores, which represent the relative abundance of each infiltrating cell in TME of each sample.

### Identification of differentially expression genes (DEGs) among distinct m6A regulator-related patterns

In our research, patients were categorized into three distinct m^6^A regulator-related patterns, according to the expression levels of 56 m^6^A regulator-related genes. Differential expression analysis of genes across these three distinct m^6^A regulator-related patterns was performed using the “limma” R package^95^. The significant threshold for identifying DEGs was set at |logFC|> 1 and an adjusted *P*-value < 0.05.

### Histological examination of the STAD TCGA samples

We acquired pathology slides of 375 GC samples through the TCGA data portal (https://portal.gdc.cancer.gov/), comprising 202 GC samples from m^6^A-related cluster 1, 120 GC samples from m^6^A-related cluster 2 and 53 GC samples from m^6^A-related cluster 3. 50 poor-quality histological sections were excluded from the analysis, leaving a total of 325 sections for further investigation (Table S14). All of these tissue slides underwent blind re-examination of diagnostic formalin-fixed paraffin-embedded slides of the tissue by a pathologist. Furthermore, semi-quantitative pathological assessment was performed on tissue slides. Pathological analysis was focused on describing the tumor immune-inflammatory response through calculating the lymphocyte scores and the stroma scores, which were representative by the percentage of fibroblasts. A 5-grade system (0–4) was applied to determine the semi-quantitative scores. Tumor purity was evaluated through histopathology by visually estimating the percentage of tumor cells among all cells on the tissue slides. Representative images of each tumor microenvironment subtype were captured at 200 × magnification using Aperio ImageScope, a pathology side-viewing software, from slides of suitable quality.

### Construction of m6A gene signature

In order to quantify the m^6^A regulator-related patterns of tumors individually, we developed a scoring system to assess the m^6^A regulator-related gene signature of GC patients, which we refer to as the m^6^A-related score. The steps to establish the m^6^A-related score are outlined below:

Initially, the m^6^A regulator-related genes (with *P* < 0.001 and cor > 0.5) were normalized across all GC samples. Subsequently, we conducted prognostic analysis for each gene, utilizing the Univariate Cox regression model. Genes that exhibited significant prognostic value in both Meta-cohort and TCGA-STAD cohort were chosen for further analysis. The m^6^A-related score of each sample was calculated using the following formula:

$${\text{m}}^{{6}} {\text{A}} - {\text{related}}\;{\text{score}}\; = \;\sum \;\left( {{\text{Coefi}}*\beta {\text{i}}} \right)$$

where Coefi represents the coefficients obtained from the Univariate Cox regression model, and βi indicates the TPM value of the corresponding m^6^A regulator-related genes.

### Relation between m6A gene signature and biological processes

We investigated the relationship between the m^6^A gene signature and various biological processes. To do so, we analyzed gene sets associated with different biological processes, including immune checkpoint, CD8 T-effector signature, antigen processing machinery and presentation, DNA damage repair mechanisms such as mismatch repair and nucleotide excision repair, DNA replication, EMT markers (EMT1, EMT2 and EMT3), angiogenesis signature, pan-fibroblast TGF beta (TGF-β) response signatures, and WNT targets^8,96,97^. Then, a correlation analysis was performed to establish the relationships between the m^6^A gene signatures and these biological processes.

### Gathering immune checkpoint blockade genomic and clinical benefit

To investigate the potential relationship between m^6^A-related scores and immunotherapy, we analyzed three immunotherapeutic cohorts. We finally selected three immunotherapeutic cohorts: advanced urothelial cancer treated with the anti-PD-L1 antibody (IMvigor210 cohort, obtained from http://research-pub.Gene.com/imvigor210corebiologies), metastatic melanoma treated with the anti-PD-1 antibody (GSE78220 cohort downloaded from GEO) and advanced malignancies treated with the anti-PD-1 and anti-CTLA4 (GSE91061 cohort downloaded from GEO) (Additional file 1: Table S1)^98,99^.

### Statistical analysis

To compare the differences between multiple groups, we conducted one-way ANOVA and Kruskal–Wallis tests. For proteomics data, GC samples were categorized into the high expression group and the low expression group based on the protein expression disparity with normal samples and the significant expression differences between normal and GC samples were analyzed by the χ^2^ test. We used the pheatmap R package to create a heatmap visualizing the relationships between m^6^A regulators and m^6^A regulator-related genes^91^. To divide the patients into high and low m^6^A-related score groups, we used the “surv-cutpoint” function from the survminer R package, based on the maximal choice of the log-rank statistics^100^. The Kaplan–Meier method was used for prognosis and survival analysis. We conducted Univariate Cox regression analysis to calculate the hazard ratio (HR) and Multivariable Cox regression analysis to select independent prognostic factors. The data from the Multivariable Cox regression analysis were visualized using the forestplot R package^101^. To investigate the mutation landscape in patients with high and low m^6^A-related score groups in TCGA-STAD cohort, we used the waterfall function of the maftools R package^91^. The result exhibited that patients with high m^6^A-related scores had a significantly lower mutation burden than patients with low m^6^A-related scores. We set the significance level at *P* < 0.005 for all statistical analyses. All processes of data manipulation were conducted by the R software (version 4.3.0) and R Bioconductor packages.

### Supplementary Information

Supplementary Information 1.

Supplementary Information 2.

## Data Availability

The datasets in this study can be found in the GEO databases (https://www.ncbi.nlm.nih.gov/geo/), TCGA databases (https://www.cancer.gov/ccg/research/genome-sequencing/tcga), Imvigor210 cohort (http://research-pub.Gene.com/imvigor210corebiologies) and PDC00024 (https://pdc.cancer.gov/pdc/study/PDC000214).
