# Supplementary material for: Comprehensive landscape of m6A regulator-related gene patterns and tumor microenvironment infiltration characterization in gastric cancer
Source: Sci Rep. 2024 Jul 16;14:16404. doi: 10.1038/s41598-024-66744-0 (PMC11252343; doi:10.1038/s41598-024-66744-0)
Supplement: Supplementary file 1 — Supplementary Information 1. [file 41598_2024_66744_MOESM1_ESM.pdf]

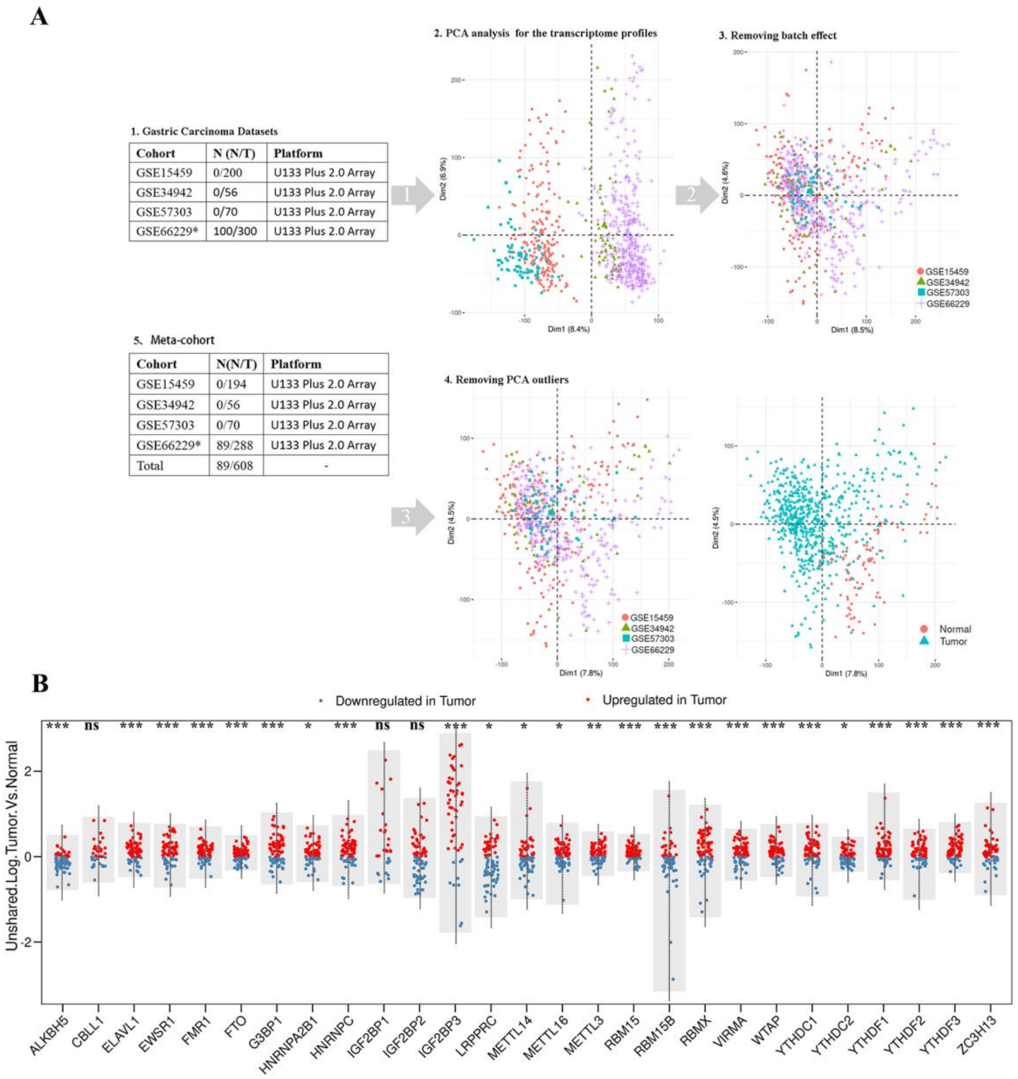

Fig. S1 A. The establishment of a Meta-cohort by PCA.

B. Protein expression levels of 27 m^{6}A regulators in the PDC000214.

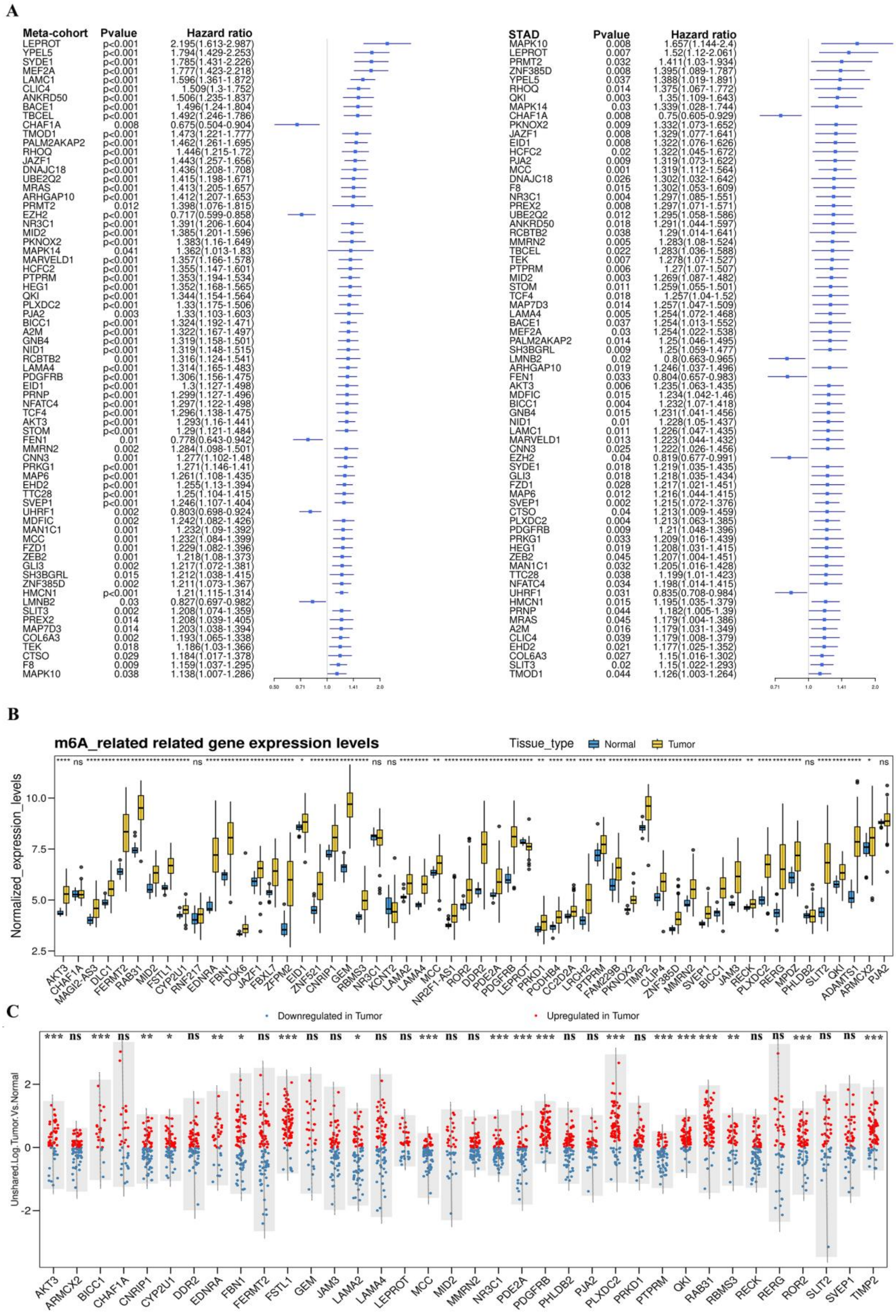

Fig. S2 A. The correlation between m^{6}A regulator-related genes and the prognosis of gastric cancer (GC) in the Meta-cohort (left) and TCGA-STAD cohort (right).

B. Expression levels of m^{6}A regulator-related genes in normal and GC

samples in the GSE54129.

C. Protein expression levels of m^{6}A regulator-related genes in the PDC000214.

A

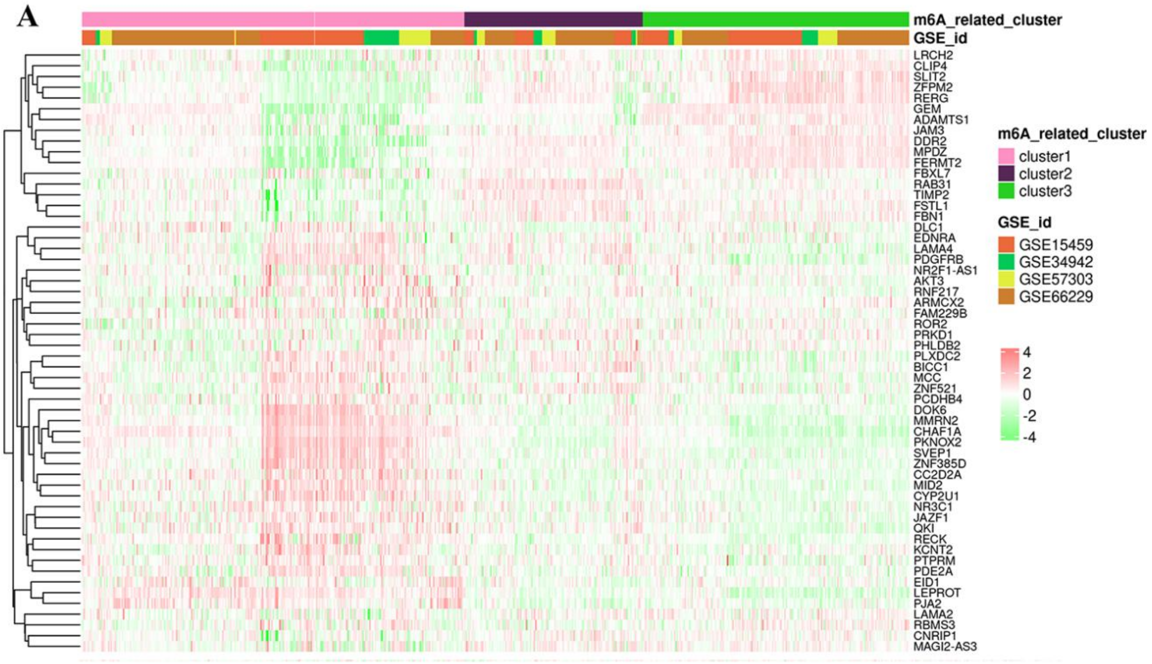

Fig. S3 A. Unsupervised clustering of 56 m^{6}A regulator-related genes in the four independent gastric cancer cohorts (GSE15459, GSE34942, GSE57303, GSE66229). The names of m^{6}A-related patterns and cohorts were applied to patient annotations.

The m^{6}A regulator-related genes were represented by each row and patients were represented by each column.

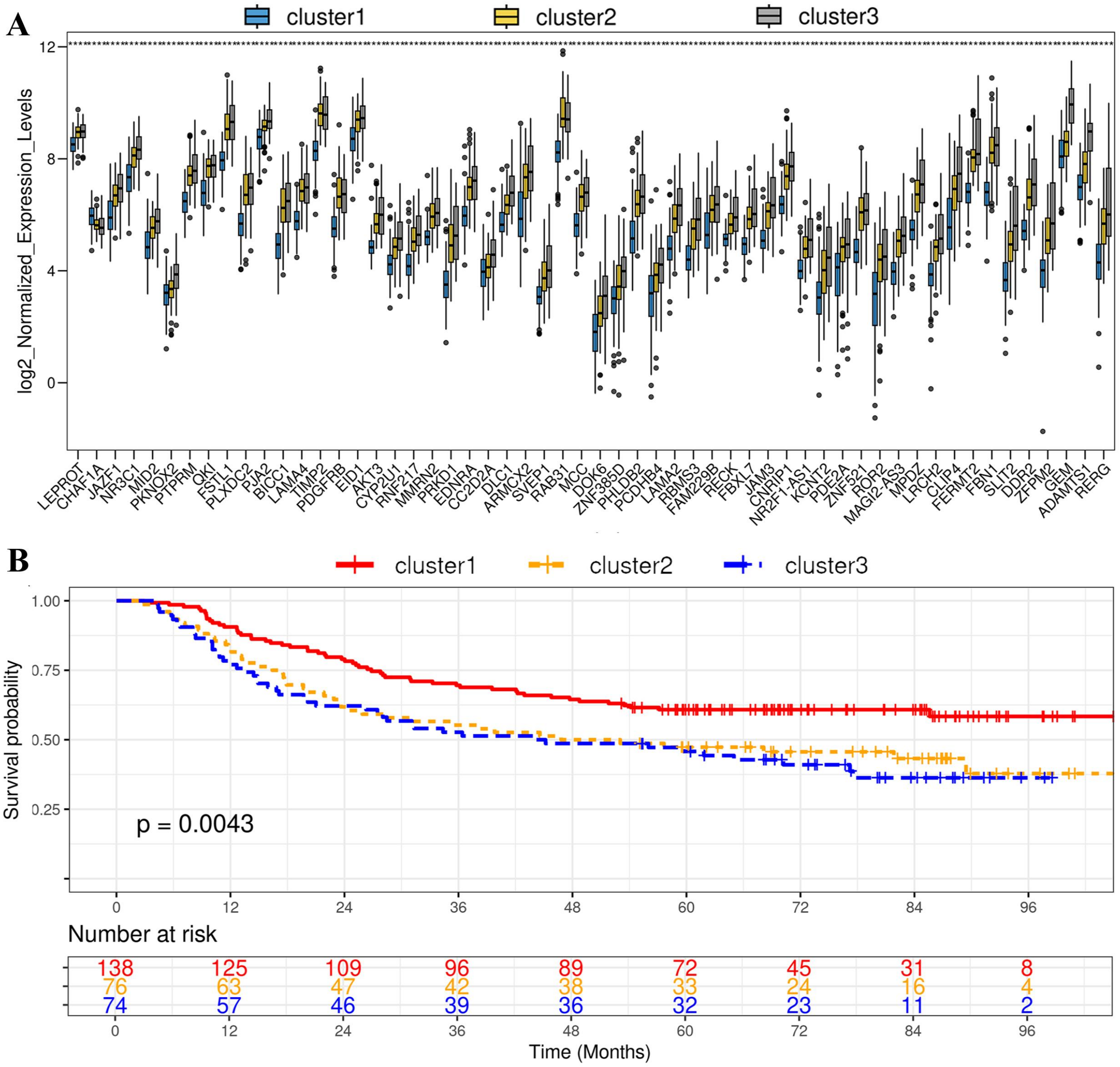

Fig. S4 A. The expression of 56 m^{6}A regulator-related genes in these three m^{6}A-related patterns. The interquartile range of values were represented by the upper and lower ends of the boxes. The median values were represented by the median lines of the boxes, and outliers were represented by the black dots. The statistical *P*-value was represented by the asterisks (**P* < 0.05; ** *P* < 0.01; *** *P* < 0.001).

B. Survival analysis for three m^{6}A-related patterns in the ACRG cohort,

included 111 cases in m^{6}A-related cluster 1, 99 cases in m^{6}A-related cluster 2 and 78 cases in m^{6}A-related cluster 3. Kaplan-Meier curves were used. The m^{6}A-related cluster 1 manifested the best overall survival. (P = 0.00051, Log-rank test).

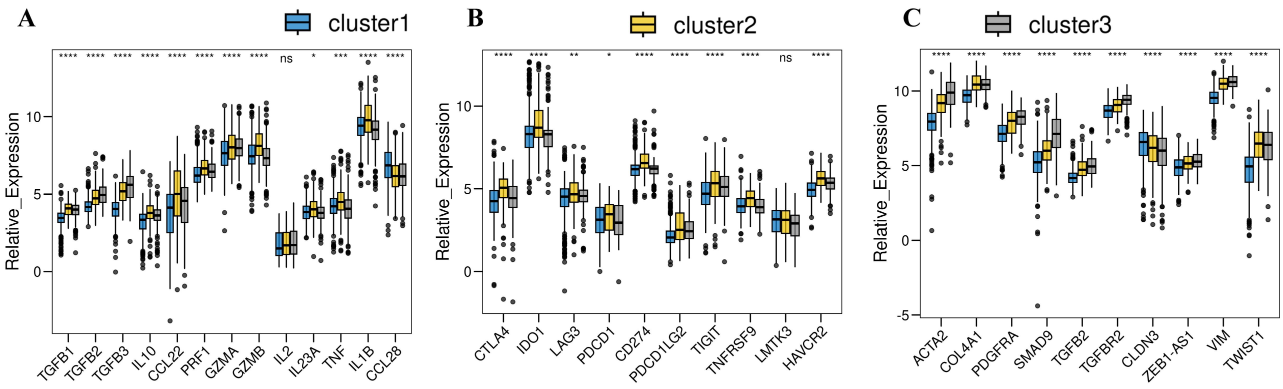

Fig. S5 A. Distinction in immune-activation-related gene expression among three m^{6}A-related patterns.

B. Distinction in immune-checkpoint-related gene expression among three m^{6}A-related patterns.

C. Distinction in the TGF-β-EMT pathway related gene expression among three m^{6}A-related patterns.

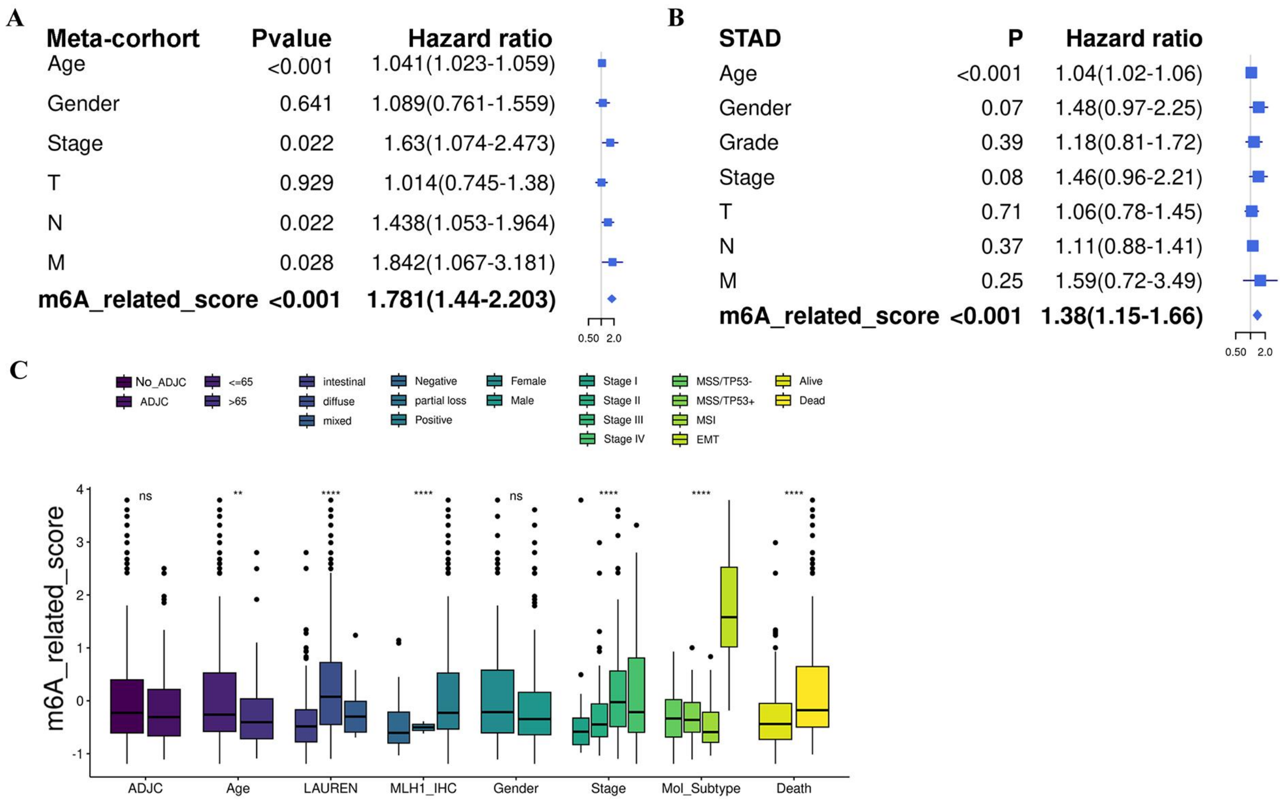

Fig. S6 A. Multivariate Cox regression analysis for m[6]A-related Score in the Meta-cohort manifested by a forest plot.

B. Multivariate Cox regression analysis for m[6]A-related Score in the TCGA-STAD cohort manifested by a forest plot.

C. Distinct in m[6]A-related Score among distinct clinical subgroups. (**P* < 0.05, ** *P* < 0.01, *** *P* < 0.001).

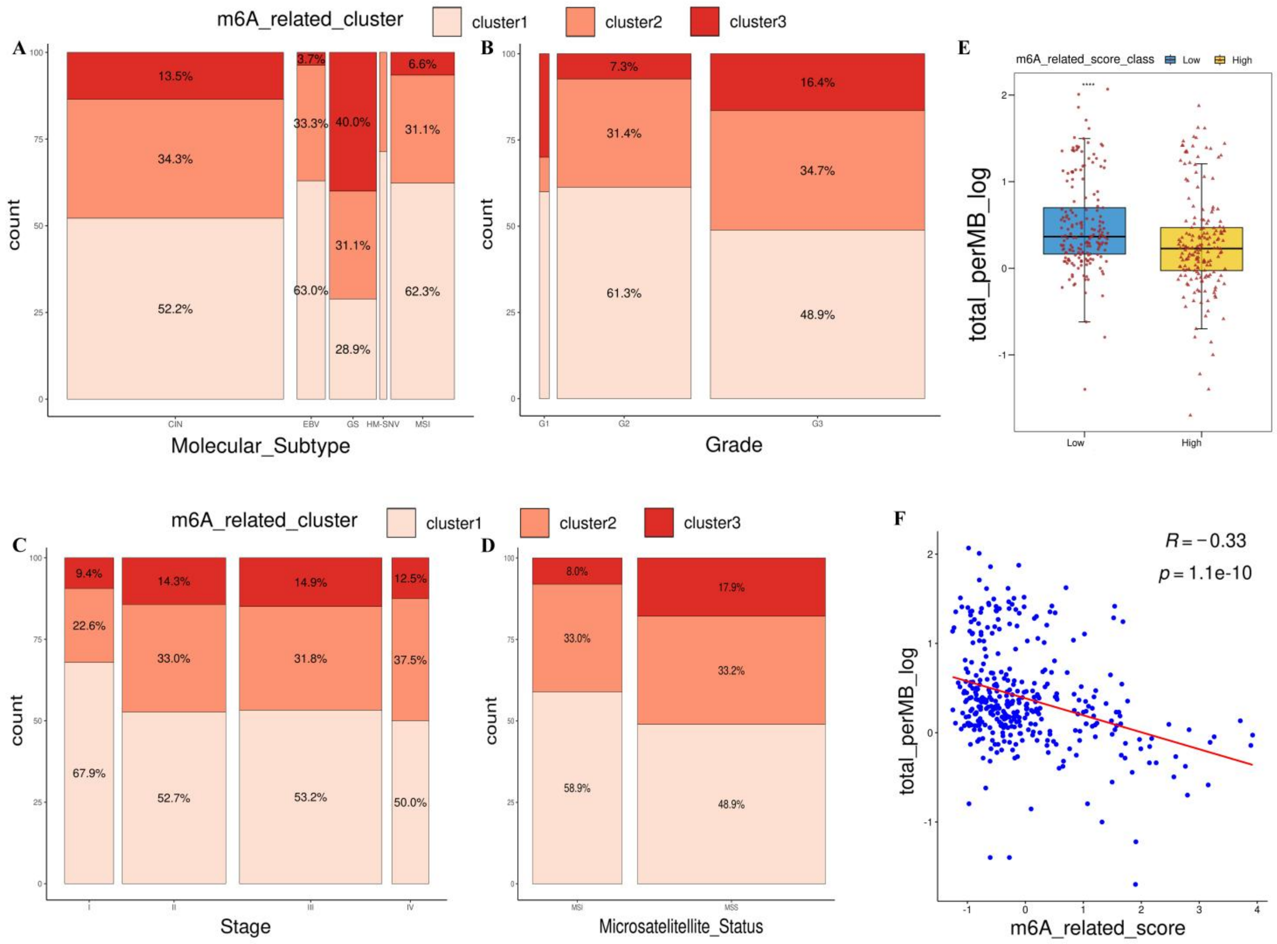

Fig. S7 A. The proportion of three m^{6}A-related patterns in the TCGA molecular subtypes.

B. The proportion of three m^{6}A-related patterns in the clinical grade.

C. The proportion of three m^{6}A-related patterns in the clinical stage.

D. The proportion of three m^{6}A-related patterns in the MSI and MSS subtypes. MSI, microsatellite instability; MSS, microsatellite stability.

E. Differences in tumor mutational burden (TMB) status between different m^{6}A-related Scores.

F. The correlation between m^{6}A-related Scores and TMB status.

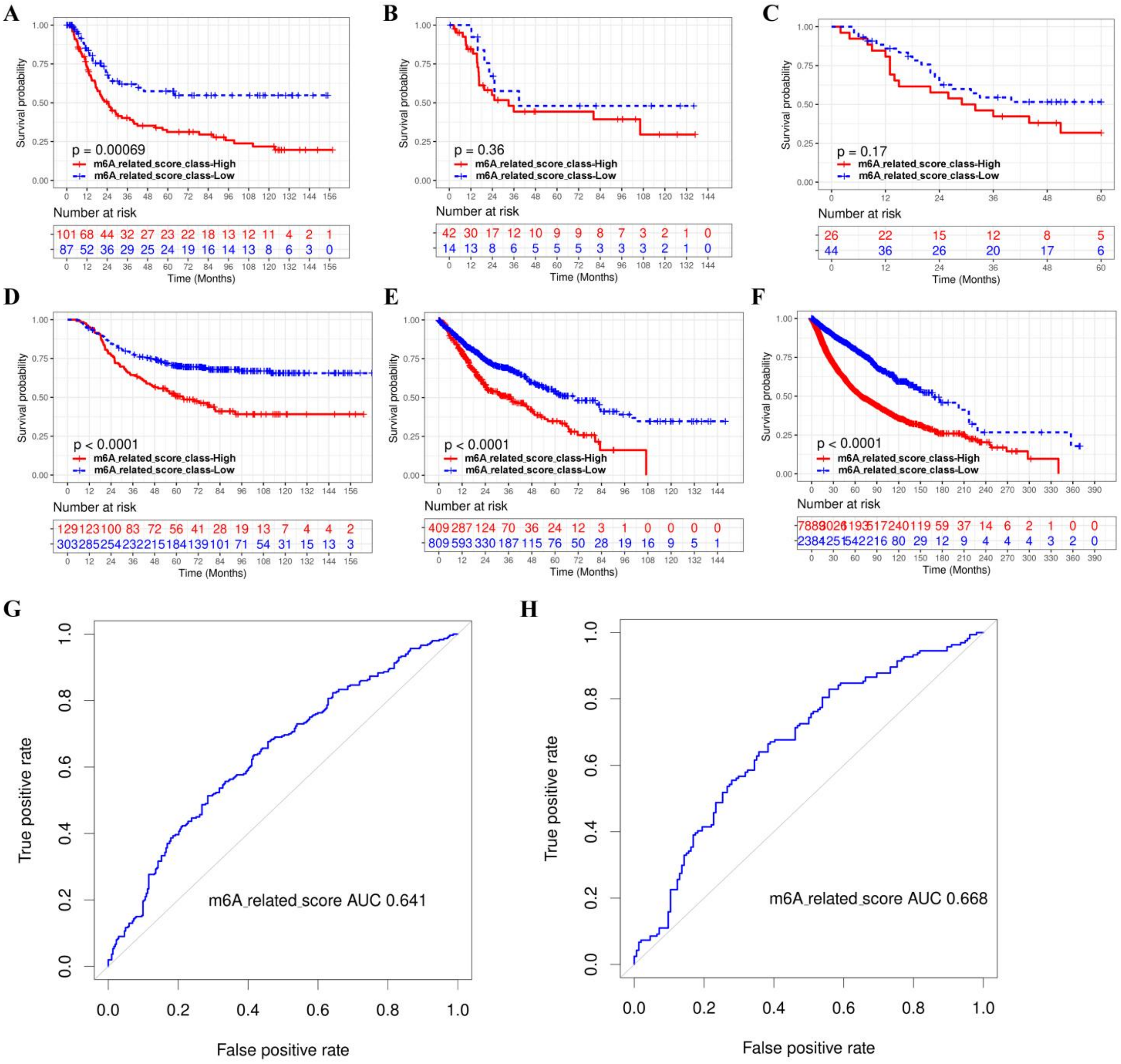

Fig. S8 A. GSE15459: HR, 2.14; 95%CI 1.43-3.22. (*P* =0.00069, Log-rank test)

B. GSE34942: HR, 1.52; 95%CI 0.67-3.46. (*P* =0.36, Log-rank test)

C. GSE57303: HR, 1.57; 95%CI 0.80-3.09. (*P* =0.17, Log-rank test)

D. GSE26253: HR, 2.00; 95%CI 1.43-2.82. (*P* < 0.0001, Log-rank test)

E. Survival analysis of Scores in all digestive cancer cohorts except GC cohorts: HR, 1.76; 95%CI 1.42-2.17. (*P* < 0.0001, Log-rank test)

F. Survival analysis of m^6A-related Scores in all cancer cohorts from TCGA. HR, 2.58; 95%CI 2.38-2.80. (*P* < 0.0001, Log-rank test)

G. The predictive value of m^6A-related Scores in all gastric cancer cohorts. AUC:

0.641.

H. The predictive value of m^6A-related Scores in elderly patients with GC. AUC: 0.667.
